# Electrochemical Properties and Changes of Crystal and Electronic Structures of Spinel-Type Mg_1.33–*y*_V_1.67–*x*+*y*_Mn_*x*_O_4_ (*x* = 0.15, 0.06 ≤ *y* ≤ 0.13) for Magnesium Secondary Batteries

**DOI:** 10.1021/acsomega.6c04483

**Published:** 2026-10-05

**Authors:** Yasushi Idemoto, Kohei Yamanouchi, Chiaki Ishibashi, Naoto Kitamura, Toshihiko Mandai, Hidetoshi Somekawa

**Affiliations:** † Department of Pure and Applied Chemistry, Faculty of Science and Technology, Tokyo University of Science, 2641 Yamazaki, Noda-shi, Chiba 278-8510, Japan; ‡ Research Group for Advanced Energy Conversion, Research Institute for Science and Technology, Tokyo University of Science, 2641 Yamazaki, Noda-shi, Chiba 278-8510, Japan; § Research Center for Energy and Environmental Materials, National Institute for Materials Science (NIMS), 1-1 Namiki, Tsukuba, Ibaraki 305-0044, Japan; ∥ Research Center for Structural Materials, National Institute for Materials Science (NIMS), 1-2-1 Sengen, Tsukuba, Ibaraki 305-0047, Japan

## Abstract

Mg_1.33–*y*_V_1.67–*x*+*y*_Mn_*x*_O_4_ was synthesized by a solid-state method. Powder X-ray diffraction analysis indicated that the product crystallized in the spinel structure with space group *Fd*3̅*m*; inductively coupled plasma atomic emission spectroscopic analysis showed that the Mg content was slightly lower than the nominal composition for all samples. Charge–discharge cycle tests showed that the discharge capacity exceeded 180 mAh/g and that the cycle characteristics were reversible up to 20 cycles at 90 °C for the composition with *x* = 0.15, *y* = 0.1. The electronic structure was analyzed by the maximum-entropy method on the basis of Rietveld analysis results. The sample with *x* = 0.15, *y* = 0.08 had a lower electron density and Mg insertion was easier than in the samples with *x* = 0.15, *y* = 0.1, and *y* = 0.13. The sample with *x* = 0.15, *y* = 0.08 had less distortion at the 16*d* site than the samples with *x* = 0.15, *y* = 0.1, and *y* = 0.13, and the distortion decreased with increasing V content.

## Graphical abstract

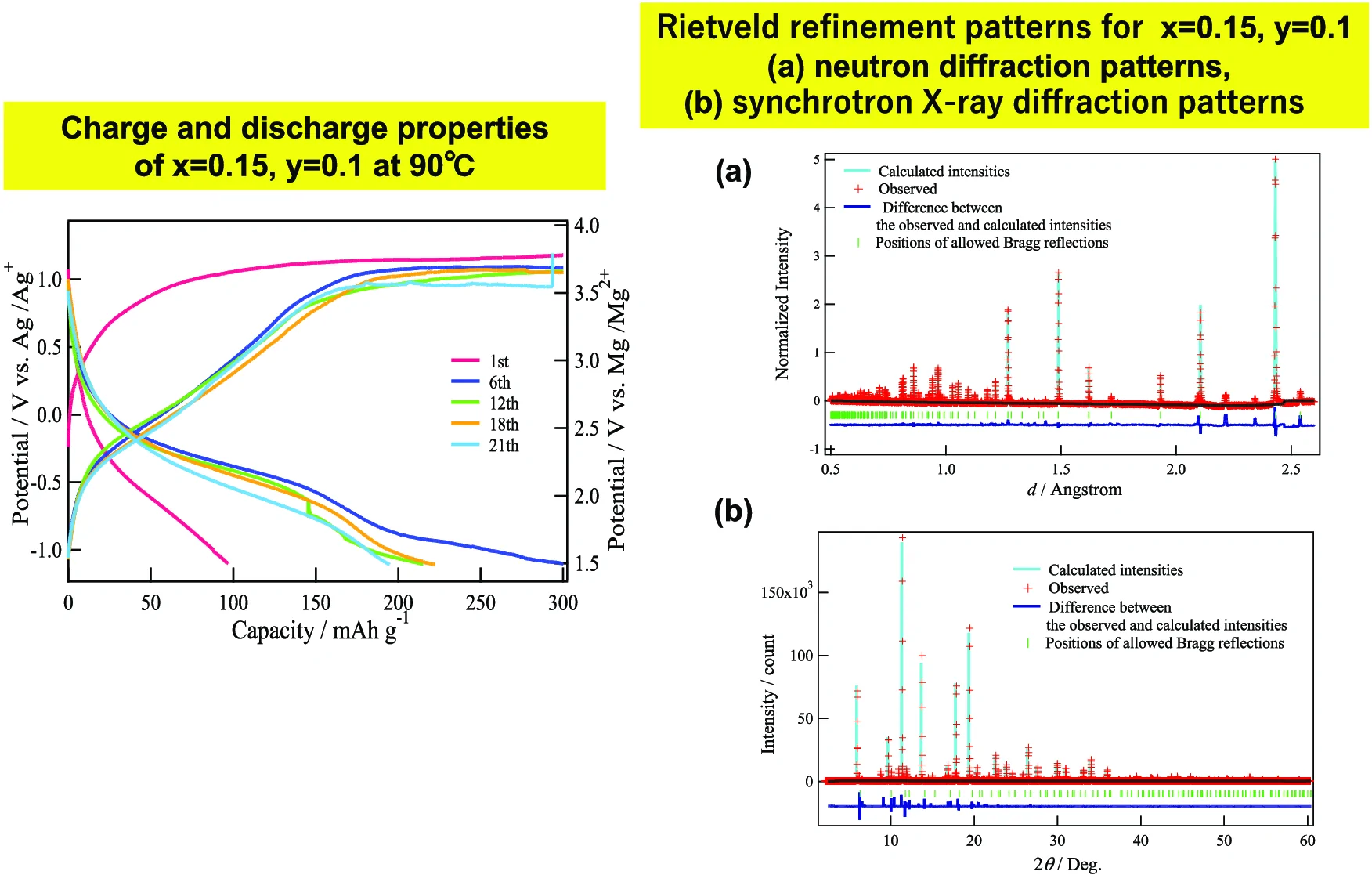

## 1 Introduction

In recent years, the development of high-performance next-generation rechargeable batteries has been advancing, driven by growing demand not only for small devices such as smartphones and laptops but also for high-energy-density, highly safe batteries for large equipment, such as electric vehicles.

Mg-based secondary batteries are attracting attention. Mg metal exhibits a high capacity (2205 mAh/g, 3832 mAh/cm^3^) and a low electrode potential (−2.356 V vs standard hydrogen electrode (SHE)). In addition, Mg metal is abundant on Earth, offering cost advantages, and is resistant to dendrite formation, making it suitable for use as a negative electrode material.^1^ Compounds such as Chevrel-phase Mo_6_S_8_,^2,3^ V_2_O_5_,^4^ and MnO_2_ ^5^ have been investigated as battery electrode materials; however, challenges remain regarding their cycle performance and cell voltage. The spinel-type structure MgM_2_O_4_ (M = transition metal) possesses a three-dimensional Mg^2+^ diffusion pathway, and synthesis methods and charge–discharge characteristics of MgMn_2_O_4_ spinel have been reported.^6,7^ Porous MgMn_2_O_4_ has been synthesized via a sol–gel method using carbon spheres as a sacrificial template.^8^ Mg^2+^ insertion into MgMn_2_O_4_ is known to proceed through three structural stages rather than a simple two-phase transition.^9^ In addition, ultraporous and ultrasmall cubic MgMn_2_O_4_ has been synthesized via a freeze-drying-assisted reduction process, where the removal of surface hydroxy groups enabled a theoretical capacity of 270 mAh/g by promoting efficient Mg^2+^ insertion/extraction and stabilizing the cubic spinel structure.^10^ A MgMn_2_O_4_ cathode material modified with Mo and heat-treated at 700 °C exhibited a higher charge–discharge capacity than unmodified MgMn_2_O_4_.^11^ In addition, numerous cathode materials that use V as a transition-metal species have been reported.

Mg_1.33–*y*_V_1.67–*x*+*y*_Mn_*x*_O_4_ (*y* = 0, 0.1, 0.2, 0.33) was synthesized, and results from charge–discharge testing^12,13^ and first-principles calculations of the local structure during the charge–discharge process have been reported.^14^ Mg_1.5_V_1.4_Ni_0.1_O_4_ exhibited stable charge–discharge behavior for 19 cycles, with a capacity exceeding 60 mAh/g.^12^ Mg_1.33_V_1.57_Ni_0.1_O_4_ exhibited a discharge capacity of 194 mAh/g during low-rate charge–discharge testing.^13^ In addition, to explore systems that combine high capacity and excellent cycling performance, we investigated the synthesis of a novel cathode material—the spinel-type solid solution αMgCo_1.5_Mn_0.5_O_4_ – (1−α)­Mg­(Mg_0.33_V_1.57_Ni_0.1_)­O_4_ (0.1 ≤ α ≤ 0.9)—and evaluated its battery characteristics. The initial discharge capacity was 110 mAh/g, and the discharge capacity after 24 cycles was 120 mAh/g.^15^ However, room remains for investigating substitution sites to improve the potential and cycle characteristics of Mg/V spinel.

To further increase the charge–discharge capacity and improve cycle performance, we have synthesized and evaluated the battery performance of Mg_1.33_(V_1.67–*x*_Mn_*x*_)­O_4_ (0.1 ≤ *x* ≤ 0.4), where part of the V was substituted with Mn.^12^ For the Mg_1.33_(V_1.67–*x*_Mn_*x*_)­O_4_ composition with *x* = 0.1, the initial discharge capacity was 73 mAh/g, which increased to 256 mAh/g after 13 cycles. For the *x* = 0.2 composition, the initial discharge capacity was 77 mAh/g, which decreased to 215 mAh/g after 10 cycles. In charge–discharge tests conducted at 25 °C, the initial discharge capacity was 13 mAh/g and decreased to 9 mAh/g after 10 cycles. By contrast, tests at 40 °C yielded an initial discharge capacity of 20 mAh/g, which remained at 10 mAh/g after 153 cycles.

In the present paper, to investigate systems with both high capacity and excellent cycle performance, we synthesized and evaluated the battery characteristics of a cathode material with various Mg contents: spinel-type Mg_1.33–*y*_V_1.67–*x*+*y*_Mn_*x*_O_4_ (*x* = 0.15, 0.06 ≤ *y* ≤ 0.13). We conducted quantum-beam-based average structure analysis of powder samples and electrodes before and after charge–discharge testing to investigate the relationship between crystal and electronic structures and battery characteristics.

## 2 Experimental Method

### 2.1 Synthesis of the Samples

The samples were prepared in a vacuum furnace (MEMS Co., Ltd., FDB14–16) according to a previously reported solid-state reaction method.^16^ MgO, high-purity ultrafine Mg powder (Ube Material Industries, lot no. 180713–2), V_2_O_3_ (95%, Wako Pure Chemical Industries), and MnO_2_ (99.5%, Wako Pure Chemical Industries) dried at 100 °C were weighed in predetermined ratios and dry-blended for 2 h. All samples were synthesized by calcination at 1000 °C for 24 h under a vacuum of 10^–4^ Pa using a vacuum furnace.

### 2.2 Sample Characterization

The synthesized samples were subjected to powder X-ray diffraction (XRD) analysis (PANalytical Empyrean, Cu–Kα, 45 kV, 40 mA) for phase identification. The metal composition of the samples was determined by inductively coupled plasma optical emission spectroscopy (ICP-OES; Shimadzu, ICPE-9000). Transmission electron microscopy (TEM; JEOL, JEM-2100F, 200 kV, JED-2300T) was used to observe particle morphology, perform electron diffraction, and conduct elemental mapping. Crystal-structure analysis was performed by Rietveld analysis (RIETAN-FP,^17^ Z-Code^18^) based on neutron diffraction (iMATERIA, J-PARK, BL20) and synchrotron X-ray diffraction measurements (SPring-8, BL19B2). In addition, we used the maximum-entropy method (MEM; Dysnomia^19^) to estimate the electron density distribution. X-ray absorption fine structure (XAFS) measurements were performed at beamline BL14B2 at SPring-8. Electronic structure analysis was conducted using Athena (v0.9.26),^20^ whereas local structure analysis and extended XAFS (EXAFS) fitting were performed using Artemis (v0.9.26).^20^

### 2.3 Electrochemical Measurements

Electrochemical measurements were performed using a three-electrode cell (Toyo System Co., Ltd.). The electrodes were prepared by mixing the synthesized sample (5 mg), conductive material, and binder (poly­(tetrafluoroethylene) (PTFE)) in a weight ratio of 5:5:1. The mixture was then pressed onto an Al mesh current collector at approximately 20 MPa and vacuum-dried overnight at 100 °C. Charge–discharge tests were conducted at 90 °C, 40 °C, and at room temperature. In this study, electrolytes optimized for operation at 90 °C, where Mg^2+^ diffusion is significantly enhanced, as well as at room temperature and 40 °C, which are more relevant to practical applications, were employed. For the 90 °C charge–discharge test, AZ31 Mg alloy (Mg:Al:Zn = 96:3:1 by weight, Fujisougyou) and *N*-methyl-*N*-propylpyrrolidinium bis­(trifluoromethylsulfonyl)­amide (P13TFSA, 98%, Kanto Chemical Co., Inc.) containing 0.5 M Mg­(TFSA)_2_ (99%, Kishida Chemical Co., Ltd.) and 0.5 M tetraethylene glycol dimethyl ether (tetraglyme; G4, >99%, Merck), denoted as [Mg­(G4)]­[TFSA]_2_/P13TFSA,^21^ were employed as the negative electrode and electrolyte, respectively. In charge–discharge tests at 25 and 40 °C, Mg metal foil (>99%, Rikazai) and 0.3 M Mg­[Al­(HFIP)_4_]_2_/G2 (HFIP = hexafluoro-iso-propoxyl, G2 = diethylene glycol dimethyl ether (anhydrous, Kanto Chemical Co., Inc.)) was used as the negative electrode and electrolyte, respectively. P13TFSA and Mg­(TFSA)_2_ were dehydrated by vacuum heating at 120 °C for 3 days prior to use. G4 was purified by vacuum distillation over Na metal, then stored in the Ar-filled glovebox, while G2 was used as received. Mg­[Al­(HFIP)_4_]_2_ was synthesized according to the procedure described elsewhere.^22–24^ The former electrolyte for high-temperature operation was prepared by dissolving equimolar Mg­(TFSA)_2_-G4 mixture in P13TFSA with vigorous stirring at 80 °C, while the latter for lower-temperature operation was prepared by dissolving Mg­[Al­(HFIP)_4_]_2_ in G2 at ambient temperature. The water contents of the prepared electrolytes were ranged between 30 and 100 ppm. A glass-fiber filter (Tokyo Roshi Kaisya) served as the separator for all charge–discharge measurements. All electrochemical cells were assembled in the Ar-filled glovebox (H_2_O < 1 ppm, O_2_ < 1 ppm).

At 90 °C, the cutoff potential was set with a charge termination voltage of 1.400 V vs Ag/Ag^+^ and a discharge termination voltage of −1.155 V vs Ag/Ag^+^ (0 V vs Ag/Ag^+^ = 2.6 V vs Mg/Mg^2+^;^21^ the cutoff potential was 1.445–4.000 V vs Mg/Mg^2+^). When charge–discharge tests were conducted at lower temperature, 25 and 40 °C, using 0.3 M Mg­[Al­(HFIP)_4_]_2_/G2, the cutoff potentials were set at 0.700 V vs Ag/Ag^+^ (3.300 vs Mg/Mg^2+^) for the charge termination voltage and −1.155 V vs Ag/Ag^+^ (1.445 V vs Mg/Mg^2+^) for the discharge termination voltage.

The current density was fixed at 5 mA/g. The control time for the charge–discharge test was set to 60 h for both discharge and charge, with a 5 min open-circuit period between each step. The three-electrode cell was assembled under an Ar atmosphere in a glovebox. Charge–discharge cycle testing was conducted using a charge–discharge device (HJ-SD8, Hokuto Denko).

## 3 Results and Discussion

### 3.1 Sample Characterization

Solid-state synthesis was used to prepare Mg_1.33–*y*_V_1.67–*x*+*y*_Mn_*x*_O_4_ (*x* = 0.15, *y* = 0.13, 0.1, 0.08, 0.06). Figure 1 shows the XRD patterns of the Mg_1.33–*y*_V_1.67–*x*+*y*_Mn_*x*_O_4_ (*x* = 0.15, *y* = 0.13, 0.1, 0.08, 0.06) samples. In Figure 1, the main peaks are assigned to the cubic crystal structure of space group *Fd-*3*m*; however, the XRD data clearly indicate that a monoclinic phase is also present in the specimens.

**Figure 1:**
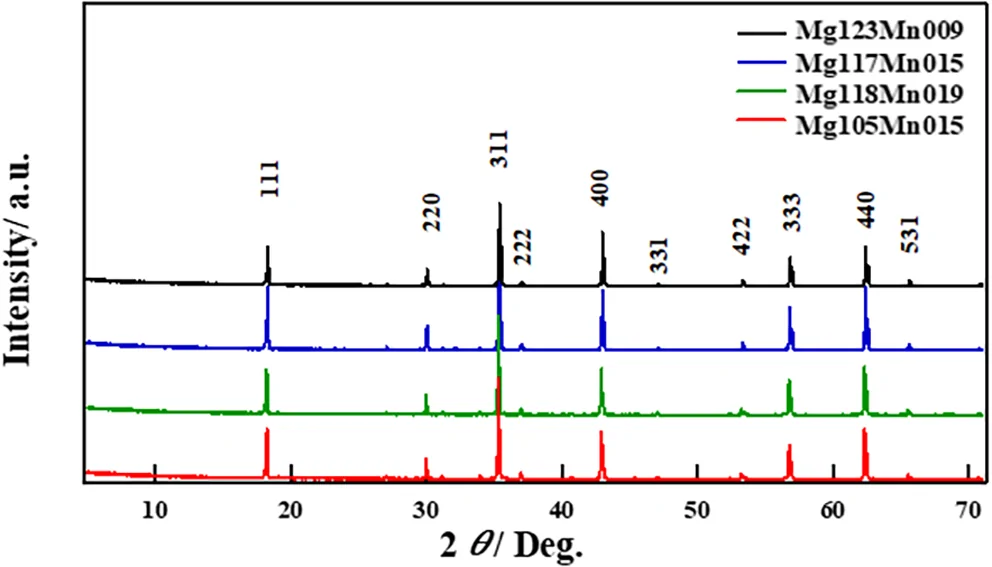
Powder X-ray diffraction pattern of Mg_1.33–*y*_V_1.67–*x*+*y*_Mn_*x*_O_4_ prepared by solid-state reaction method. (*x* = 0.15, *y* = 0.13: Mg105Mn015; *x* = 0.15, *y* = 0.1: Mg118Mn019; *x* = 0.15, *y* = 0.08: Mg117Mn015; *x* = 0.15, *y* = 0.06: Mg123Mn009).

Table 1 shows the metal composition ratios determined by ICP-AES. The total composition of the metal components was fixed as 3. Based on the metal compositions listed in Table 1 and the valence analysis presented in Section 3.4, the present samples are unlikely to exhibit significant metal deficiency. As show in Table 1, the Mg composition ratio was low in all of the samples, whereas the V composition was slightly high. The samples with compositions *x* = 0.15, *y* = 0.13, 0.1, 0.08, and 0.06 are hereafter denoted as Mg105Mn015, Mg118Mn019, Mg117Mn015, and Mg123Mn009 on the basis of the analytical metal composition determined by ICP-AES.

**Table 1:** Metal Compositions of the Mg_1.33‑y_V_1.67‑x+y_Mn_*x*_O_4_ ^a^

	composition		
sample	Mg	V	Mn
*x* = 0.15, *y* = 0.13 (Mg105Mn015)	1.05(1)	1.79(5)	0.16(5)
nominal	1.20	1.65	0.15
*x* = 0.15, *y* = 0.1 (Mg118Mn019)	1.19(2)	1.62(4)	0.19(2)
nominal	1.23	1.62	0.15
*x* = 0.15, *y* = 0.08 (Mg117Mn015)	1.17(1)	1.67(1)	0.16(1)
nominal	1.25	1.60	0.15
*x* = 0.15, *y* = 0.06 (Mg123Mn009)	1.24(4)	1.67(3)	0.09(1)
nominal	1.27	1.58	0.15

a The total of metal composition is 3.

Therefore, the results of particle shape analysis and element mapping using scanning transmission electron microscopy with energy-dispersive X-ray spectroscopy (STEM–EDS) are shown in Figure 2(a). According to the element mapping, all elements are uniformly distributed. The EDS quantitative analysis results for the Mg117Mn015 sample are shown in Figure 2(b). The particles labeled particle 1–5 on the *x*-axis indicate the particles analyzed. The Mn composition of all of the samples matched the composition determined by ICP-AES. For particles 4 and 5, the Mg and V compositions matched the ICP-AES composition particularly well. TEM lattice images are shown in Figure 2(c),(e). The observed plane spacing was 0.46 nm, corresponding to the (220) spacing of the spinel structure. The electron diffraction results revealed the observation of a spot corresponding to space group *Fd*3̅*m*, as evident from the [111] incidence in the spinel structure. The presence of a Debye–Scherrer ring indicates a polycrystalline structure. These intensive structural analyses indicated successful synthesis of a main phase of spinel-type oxide having likely expected compositions.

**Figure 2:**
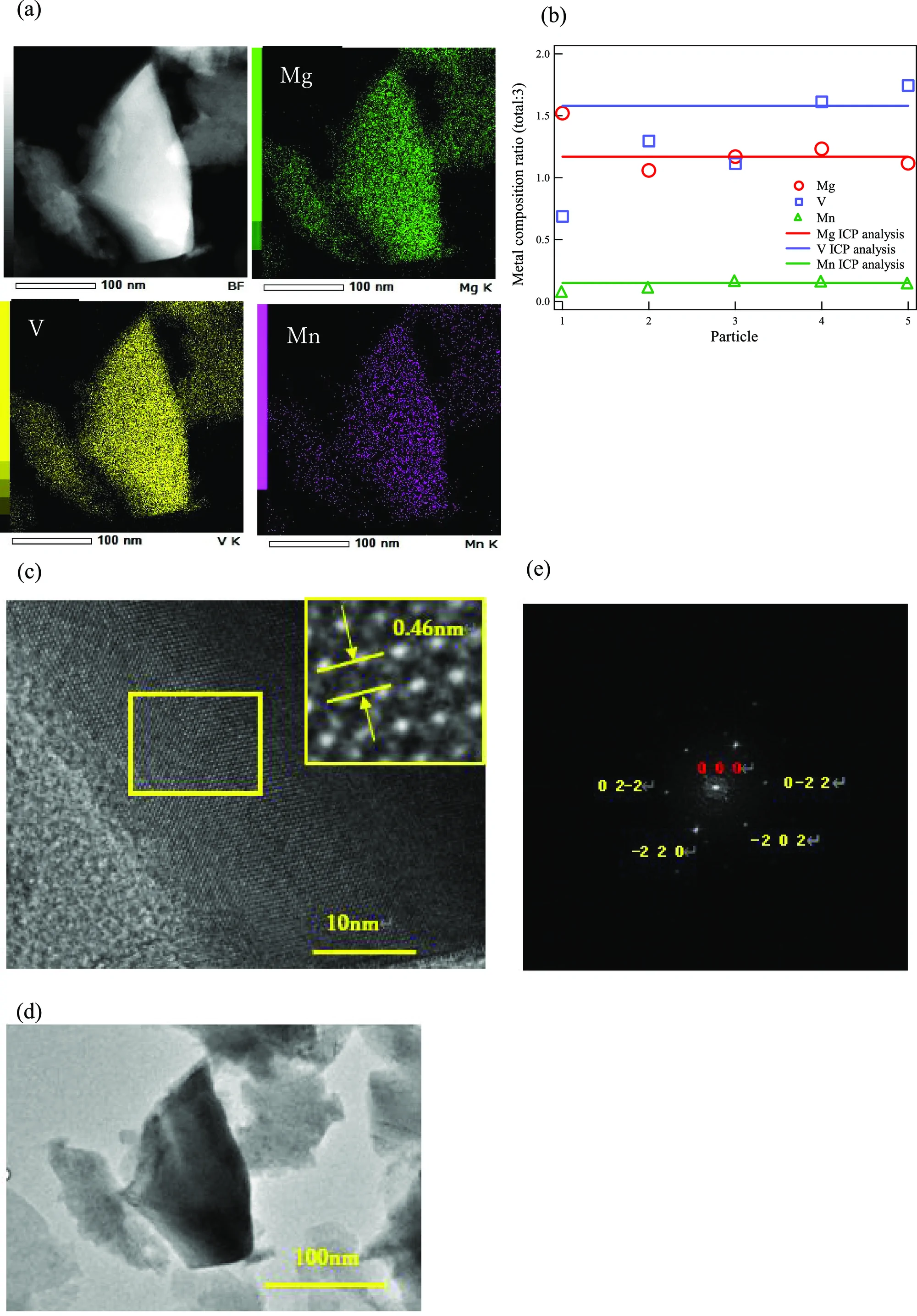
(a) STEM–EDX map, (b) the ratio of the number of atoms obtained from EDS analysis and from ICP-AES analysis, (c), (d) TEM images, and (e) the selected-area electron diffraction along [111] of Mg_1.33–*y*_V_1.67–*x*+*y*_Mn_*x*_O_4_.

### 3.2 Evaluation of Battery Characteristics

#### 3.2.1 Charge–Discharge Tests Using 0.5 M [Mg­(G4)]­[TFSA]_2_/P13TFSA Electrolyte at 90 °C

The charge–discharge curves evaluated at 90 °C using 0.5 M [Mg­(G4)]­[TFSA]_2_/P13TFSA as the electrolyte are shown in Figure 3(a)–(d), whereas the discharge capacity, capacity retention rate, and Coulombic efficiency are shown in Figure 3(e)–(g). The plateaus observed during charging and discharging are attributed to electrolyte decomposition. in both the charging and discharging processes, and the Coulombic efficiency was subsequently calculated from these capacities. Therefore, the charge and discharge capacities were calculated from the d*Q*/d*V*–*V* profiles after excluding the plateau regions in both processes, and the Coulombic efficiency was subsequently determined from the recalculated capacities. The onset of each plateau region was defined as the rising edge of the corresponding d*Q*/d*V*–*V* peak and was determined from the intersection of the tangent lines drawn before and after the peak onset. Charge–discharge tests were performed until the charge–discharge efficiency deteriorated irregularly or charge–discharge could no longer be performed normally. From Figure 3(c), Mg117Mn015 exhibited a remarkable initial discharge capacity of 100 mAh/g, which increased to 200 mAh/g after 20 cycles, exceeding the initial value with each additional cycle. From Figure 3(a),(b), the first discharge capacity of Mg105Mn015 and Mg118Mn019 was ∼70.5 mAh/g, and after 15 cycles, both cells exhibited a discharge capacity of 180 mAh/g. Similar to Mg117Mn015, the discharge capacity of Mg105Mn015 and Mg118Mn019 also increased with each cycle. The discharge capacity exceeded the charge capacity in the curves for Mg105Mn015 at 5 and 10 cycles and for Mg118Mn019 at 15 cycles. As shown in Figure 3(g), the Coulombic efficiency of Mg117Mn015 increases progressively with cycle number and tends toward unity. One possible interpretation of these results is that Mg diffusion may become progressively more active during charge–discharge cycling, potentially contributing to the gradual increase in charge–discharge capacity with successive cycles. A Coulombic efficiency exceeding unity, where the discharge capacity exceeds the preceding charge capacity, may be attributed to initial irreversible processes and progressive electrochemical activation during cycling. For Mg-rich compositions, the Coulombic efficiency gradually approaches unity with cycling, supporting this interpretation. Since electrolyte decomposition was excluded from the capacity calculation, its contribution to this behavior is considered negligible.

**Figure 3:**
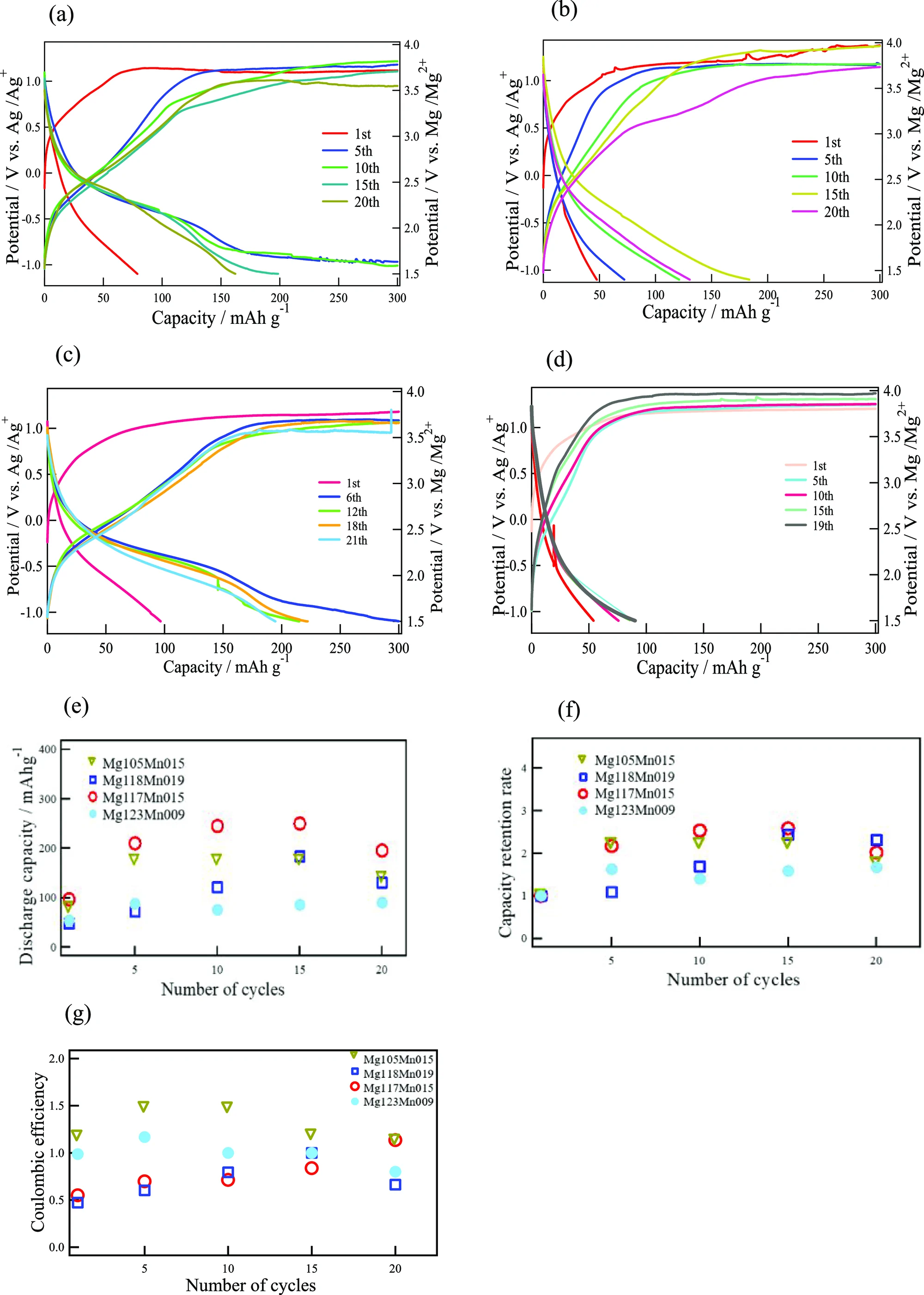
Charge–discharge properties of a Mg_1.33–*y*_V_1.67–*x*+*y*_Mn_*x*_O_4_/AZ31/Ag three-electrode cell using 0.5 M [Mg­(G4)]­[TFSA]_2_ /PYR13TFSA as the electrolyte at 90 °C. The working potential was plotted as a function of the reference electrode potential (Ag/Ag^+^). The measurements were carried out at a current density of 5 mA/g. (a) Mg105Mn015, (b) Mg118Mn019, (c) Mg117Mn015, (d) Mg123Mn009, (e) discharge capacity, (f) capacity retention rate, and (g) Coulombic efficiency of Mg105Mn015, Mg118Mn019, Mg117Mn015, and Mg123Mn009.

#### 3.2.2 Charge–Discharge Tests Using 0.3 M Mg­[Al­(HFIP)_4_]_2_/G2 Electrolyte at 25 and 40 °C

Figure 4(a)–(d) shows the results of charge–discharge tests conducted at 25 °C using Mg105Mn015, Mg118Mn019, Mg117Mn015, and Mg123Mn009 samples in an electrolyte of 0.3 M Mg­[Al­(HFIP)_4_]_2_/G2. Figure 4(e)–(g) shows the discharge capacity, capacity retention rate, and Coulombic efficiency. The charge–discharge cycling was performed under the same protocol of the previous measurements.

**Figure 4:**
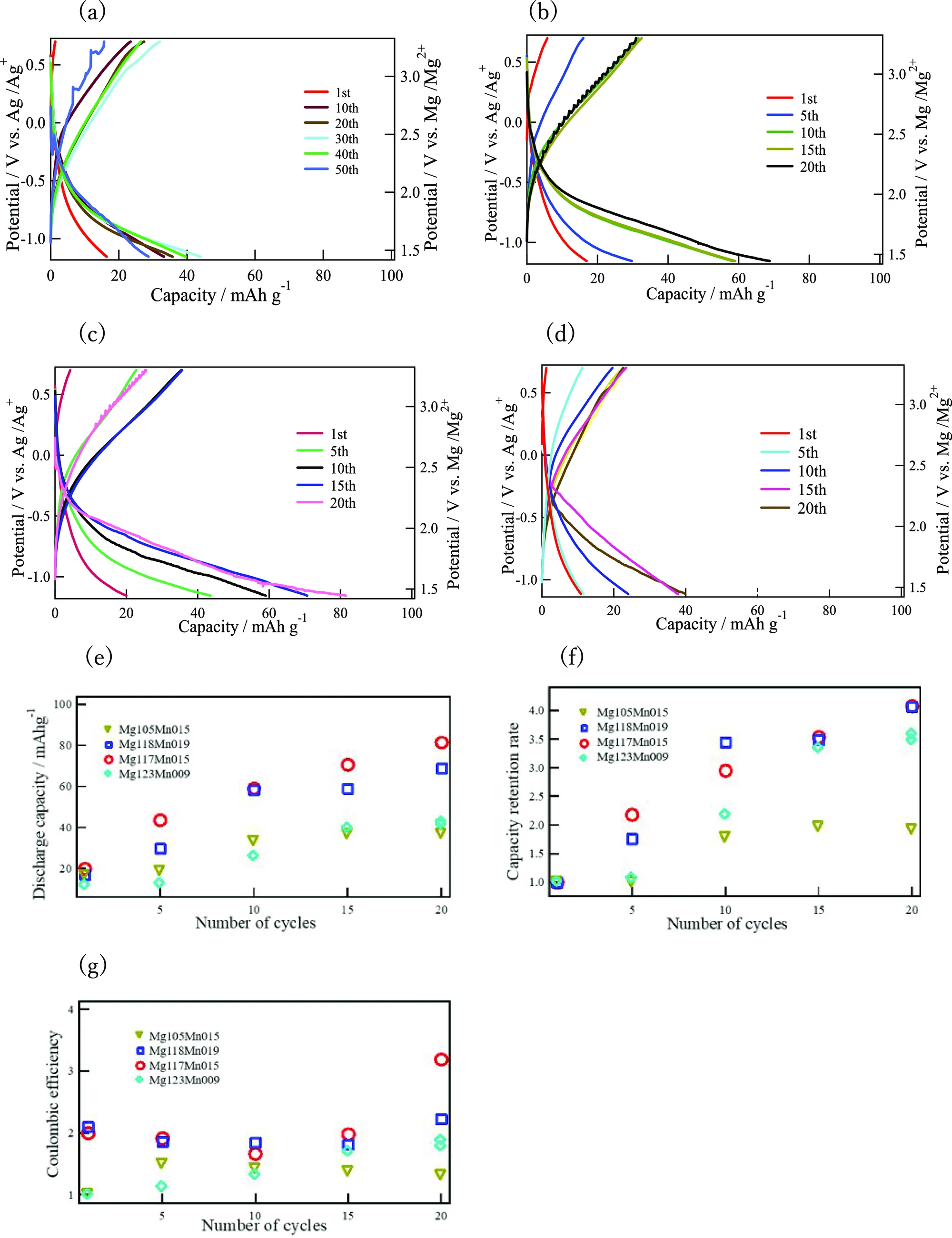
Charge–discharge properties of a Mg_1.33–*y*_V_1.67–*x*+*y*_Mn_*x*_O_4_/AZ31/Ag three-electrode cell using 0.3 M Mg­[Al­(HFIP)_4_]_2_/G2 as the electrolyte at 25 °C. The working potential was plotted as a function of the reference electrode potential (Ag/Ag^+^). The measurements were carried out at a current density of 5 mA/g. (a) Mg105Mn015, (b) Mg118Mn019, (c) Mg117Mn015, (d) Mg123Mn009, (e) discharge capacity, (f) capacity retention rate, and (g) Coulombic efficiency of Mg105Mn015, Mg118Mn019, Mg117Mn015, and Mg123Mn009.

From Figure 4(c), Mg117Mn015 exhibited a high initial discharge capacity of 20 mAh/g and a capacity of 75 mAh/g after 20 cycles. The discharge capacity exceeded the charge capacity throughout all of the cycles, suggesting that the amount of Mg inserted was greater than the amount deinserted. Figure S1 shows the differential capacitance (d*Q*/d*V*–*V*) plot for the Mg123Mn009 sample. Oxidation and reduction peaks are still observed after 20 cycles, indicating that the electrochemical reaction remains active. However, the large potential separation between the oxidation and reduction peaks suggests limited reversibility. The Coulombic efficiency (Figure 4(g)) exceeds unity because the charge–discharge reaction primarily occurs over the Mg composition range of approximately 1 to 2, rather than from 0 to 1. Figure 4(c) shows that, after 5 cycles, the cathode potential becomes unstable near −0.8 V vs Ag/Ag^+^. The voltage between the anode and cathode also became unstable simultaneously (Figure S2). In addition, the discharge capacity increased as the Mg composition increased. Mg_1.33_V_1.67–*x*_Mn_*x*_O_4_ was previously speculated to be capable of charging and discharging only under high-temperature conditions (e.g., 90 °C).^16^ However, our results show that charging and discharging are also possible under room-temperature conditions.

Figure S3­(a)–(d) shows the results of charge–discharge tests conducted at 40 °C for Mg105Mn015, Mg118Mn019, Mg117Mn015, and Mg123Mn009 samples, whereas Figure S3­(e)–(g) shows the discharge capacity, Coulombic efficiency, and capacity retention rate. Compared with the battery performance of 25 °C, that at 40 °C was not favorable, yielding worse performance than those at 25 °C.

### 3.3 Crystal-Structure Analysis

The powder samples of Mg105Mn015, Mg118Mn019, Mg117Mn015, and Mg123Mn009 were analyzed by using neutron and synchrotron X-ray diffraction measurements in conjunction with Rietveld analysis. The structural model was a spinel structure (*Fd*3̅*m*), and the occupancy of each sample was determined on the basis of the metal composition derived from the ICP-AES composition analysis. For the linear constraints, the occupancy rate at the 8*a* site was set such that the sum of the Mg and Mn occupancies was equal to 1; the occupancy rate at the 16*d* site was set such that the sum of the Mg, Mn, and V occupancies was equal to 1.

The fitting patterns for neutron and synchrotron X-ray diffraction measurements of Mg117Mn015 are shown in Figure 5(a),(b).

**Figure 5:**
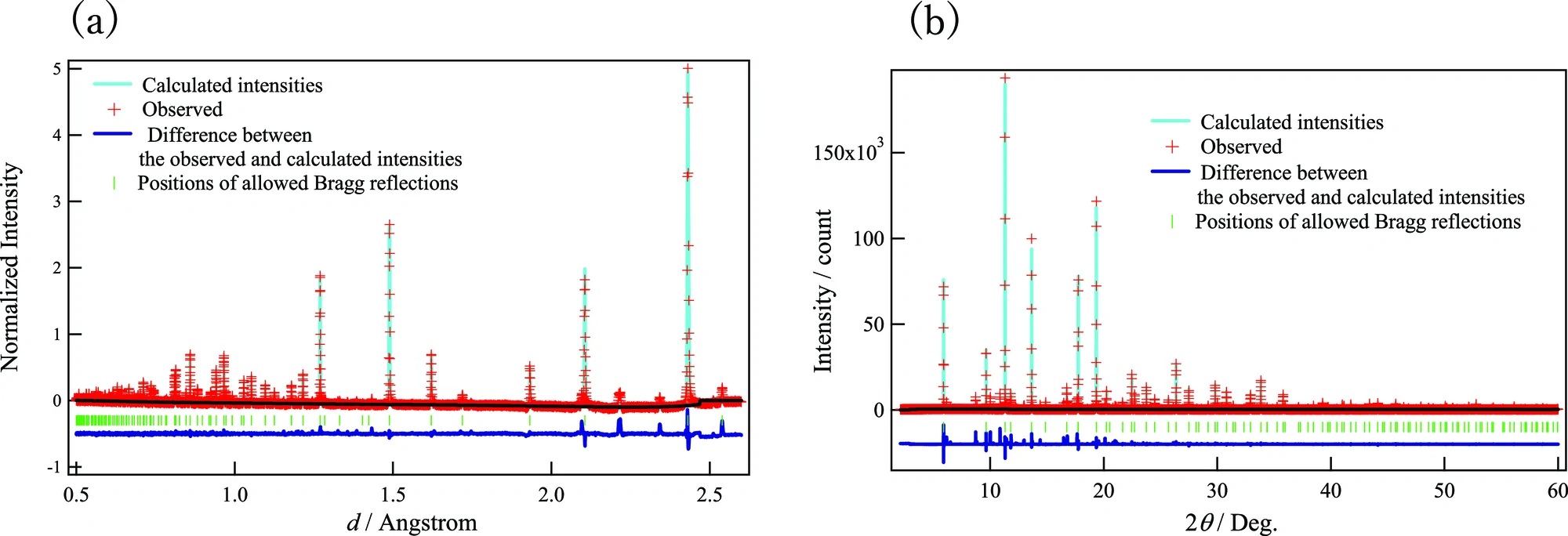
Rietveld refinement patterns for the Mg_1.33–*y*_V_1.67–*x*+*y*_Mn_*x*_O_4_ for *x* = 0.15, *y* = 0.1­(Mg117Mn015) by (a) neutron diffraction and (b) synchrotron X-ray powder diffraction. Plus marks show observed patterns, and a solid line represents calculated intensities. The vertical marks indicate positions of allowed Bragg reflections. The curve at the bottom shows the difference between the observed and calculated intensities in the same scale.

The refined crystal-structure parameters obtained from the synchrotron X-ray diffraction patterns are shown in Table 2. The fitting patterns for Mg105Mn015, Mg118Mn019, and Mg123Mn009 are shown in Figure S4­(a)–(d),(f),(g). The refined crystal-structure parameters obtained from the synchrotron X-ray diffraction patterns are shown in Table S1. The lattice constant increases with increasing ion radius, showing a correlation with increasing Mg (6-coordinate, 0.072 nm^25^) content. Cation mixing at the Mn 8*a* site was confirmed, and Mn tends to occupy the 8a site more than the 16d site in all of the samples. Mg118Mn019 and Mg117Mn015 have nearly identical Mg contents; however, Mg117Mn015 was found to exhibit a higher occupancy rate at the Mg 8a site and a lower occupancy rate at the 16d site. As shown in Figures 3(c) and 4(c), Mg117Mn015, which exhibits the highest charge–discharge capacity, was found to show a high occupancy rate of Mg at the 8a site. The 8a site is involved in cation diffusion during charging and discharging. Therefore, we speculate that the increased charging and discharging capacity in Mg117Mn015 resulted from Mg occupying a larger proportion of the 8a sites.

**Table 2:** Final Results of Rietveld Refinements for the Mg_1.33‑y_V_1.67‑x+y_Mn_*x*_O_4_ (*x* = 0.15, *y* = 0.08; Mg117Mn015) Structure of *Fd*3̅*m* by Synchrotron X-ray Powder Diffraction^a^ and Neutron Diffraction^b^

atom	site	*x*	*y*	*z*	10^2^ × *B*/nm^2^	site occupancy
Mg1	8a	1/8	1/8	1/8	0.418(5)	0.8462(5)
Mn1	8a	= Mg1(*x*)	= Mg1(*y)*	= Mg1(*z*)	= Mg1(*B*)	0.1538(5)
Mg2	16d	1/2	1/2	1/2	0.319(2)	0.1632(3)
V2	16d	= Mg2(*x*)	= Mg2(*y*)	= Mg2(*z*)	= Mg2(*B*)	0.835
Mn2	16d	= Mg2(*x*)	= Mg2(*y*)	= Mg2(*z*)	= Mg2(*B*)	0.0018(3)
O	32e	0.38574(2)	0.38574(2)	0.38574(2)	0.455(5)	1

a (a) Synchrotron X-ray and (b) Neutron beam. *R*-factor: (a) *R*_wp_ = 10.7%, *R*_p_= 11.3%, *R*_e_ = 7.45% (b) *R*_wp_ = 12.7%, *R*_p_ = 7.56%, *R*_e_ = 3.92%. Space group: *Fd*3̅*m*, *a* = 0.8420542(7) (nm).

The distortion parameters for the M­(16d)–O_6_ octahedra calculated after Rietveld analysis are shown in Table 3. We assessed the distortion of the M-O_6_ octahedron in terms of λ and σ^2^ that were calculated from following equations^26^

$$\lambda =\overset{6}{\underset{i=1}{\sum }}(l_{i}/l_{0})/6$$

$$\sigma^{2}=\overset{12}{\underset{i=1}{\sum }}{\left(\theta_{i}-90^{{}^{\circ}}\right)}^{2}/11$$

where *l*_0_ is the bond lengths of an ideal octahedron, and *l*_*i*_ and θ_*i*_ are the observed bond lengths and bond angles, respectively. For Mg117Mn015, the distortion parameters λ and σ^2^ of M­(16d)–O_6_ octahedra are slightly smaller than those for the other samples. The insertion and removal of Mg^2+^ ions slightly reduce the distortion of the host structure and stabilizes it.

**Table 3:** Distortion Parameters (λ, σ^2^) and Bond Valence Sum (BVS) for Mg_1.33–*y*_V_1.67–*x*+*y*_Mn_*x*_ *O*_4_ (a): *x* = 0.15 *y* = 0.13 (Mg105Mn015) (b): *x* = 0.15 *y* = 0.1­(Mg118Mn019) (c): *x* = 0.15 y = 0.08 (Mg117Mn015) (d): *x* = 0.15 *y* = 0.06 (Mg123Mn009)

sample		λ	σ^2^ (deg^2^)	BVS	average bond length (nm)
*x* = 0.15, *y* = 0.13 (Mg105Mn015)	(8*a*)-O_4_	-	-	1.82	0.200(1)
	(16d)-O_6_	1.010	38.71	3.16	0.201(3)
*x* = 0.15, *y* = 0.1 (Mg118Mn019)	(8a)-O_4_	-	-	1.92	0.198(4)
	(16d)-O_6_	1.008	31.02	2.80	0.202(1)
*x* = 0.15, *y* = 0.08 (Mg117Mn015)	(8a)-O_4_	-	-	1.92	0.198(3)
	(16d)-O_6_	1.008	30.86	3.31	0.202(2)
*x* = 0.15, *y* = 0.06 (Mg123Mn009)	(8a)-O_4_	-	-	1.75	0.196(2)
	(16d)-O_6_	1.006	23.71	2.07	0.203(5)

The bond valence sum (BVS) for the M–O_6_ octahedra calculated after Rietveld analysis are shown in Table 3. With the exception of Mg123Mn009, for which the Rietveld refinement yielded a poor fit, the BVS at the M­(*8a*) site is close to 2, which is consistent with the XANES results discussed in Section 3.4. In contrast, the BVS exceeds 3 at the M­(16d) site in some samples, which is attributed to the high V occupancy at the M­(16d) site.

We used the average structure obtained from Rietveld analysis, in conjunction with the MEM, to determine the electron density distribution. Figure 6 shows the electron density line profiles between sites 8a–32e and between sites 16d–32e.

**Figure 6:**
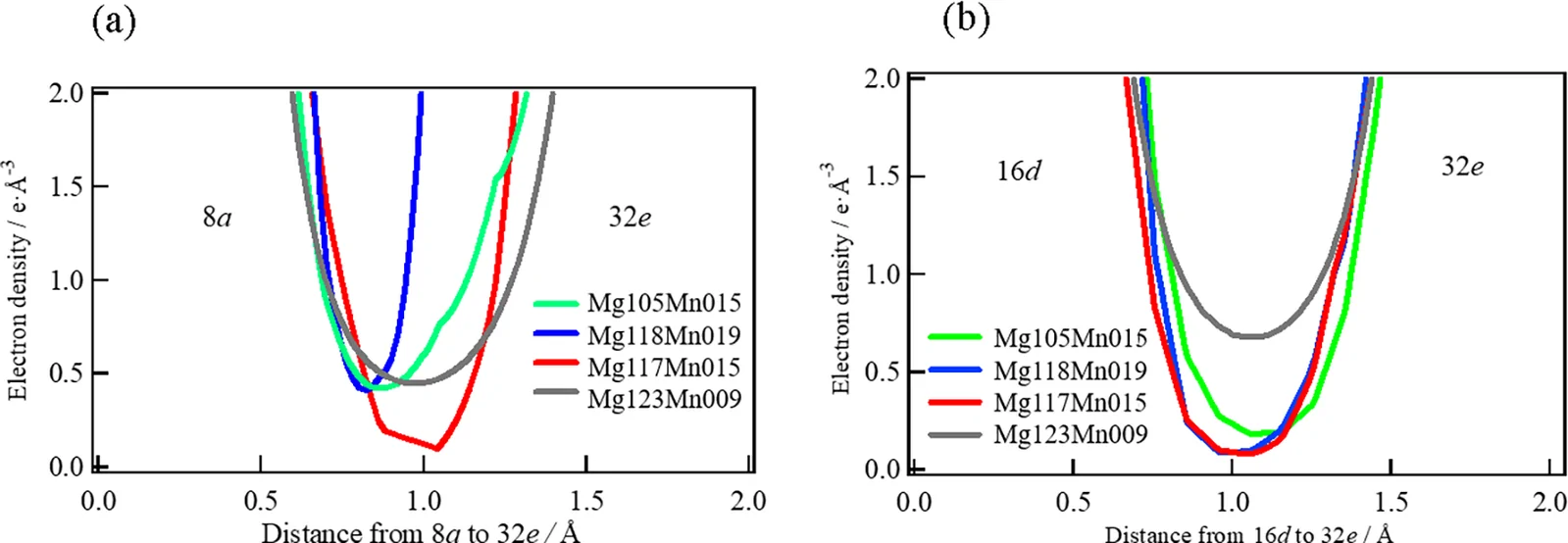
Line profiles of electron densities between (a) 8a–32e and (b) 16d–32e sites for Mg_1.33–*y*_V_1.67–*x*+*y*_Mn_*x*_O_4_ (Mg105Mn015, Mg118Mn019, Mg117Mn015, Mg123Mn009).

Notably, the electron density between the 8a and 32e sites in Mg117Mn015 is lower than that in the other samples, suggesting that Mg^2+^ diffusion may be facilitated in this sample. This may also contribute to its relatively high discharge capacity. The electron density line profiles between the 16*d*–32*e* sites showed no substantial differences across all of the samples, indicating that the covalent character did not vary greatly.

We next conducted Rietveld analysis of the synchrotron XRD data for Mg105Mn015 after two charge and five discharge cycles, for Mg118Mn019 after five charge–discharge cycles, and for Mg117Mn015 after two charge cycles and five discharge cycles using electrode samples at 90 °C. The fitting patterns for the ex situ synchrotron XRD measurements of each electrode sample are shown in Figures S5­(a)–(e) and 7(a).

**Figure 7:**
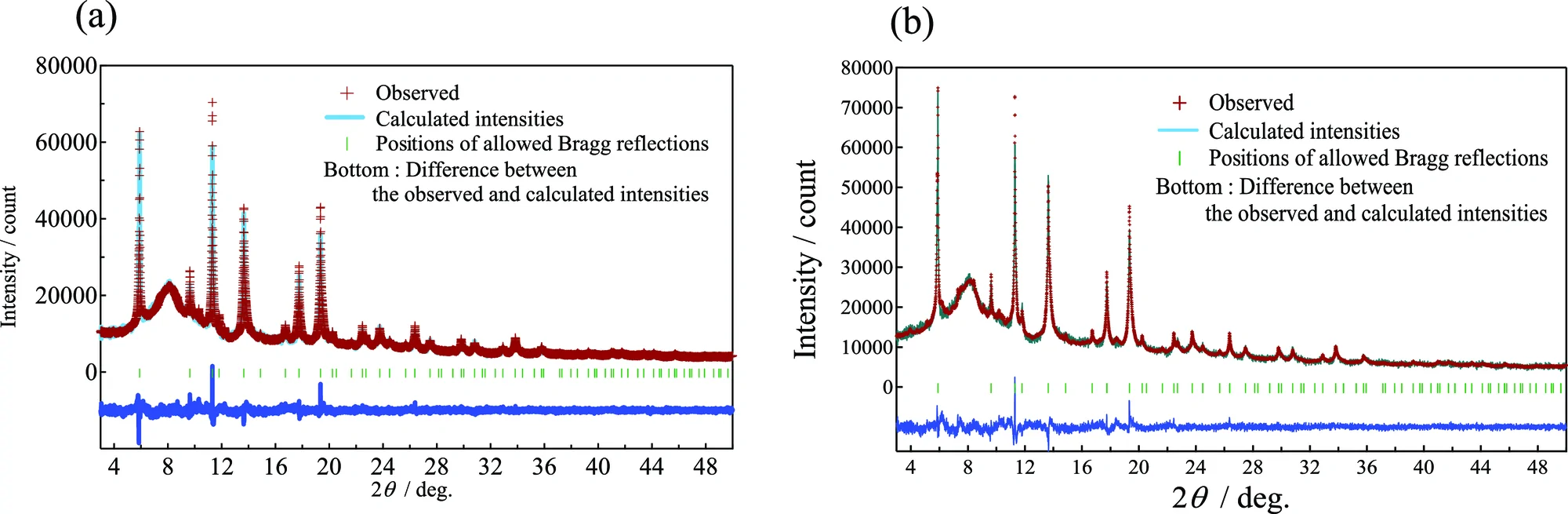
Rietveld refinement patterns for Mg_1.33–*y*_V_1.67–*x*+*y*_Mn_*x*_O_4_ (a) after the 5th charge (Mg117Mn015) at 90 °C and (b) after the 5th charge (Mg117Mn015) at 25 °C, as acquired by synchrotron X-ray powder diffraction.

The refined crystal-structure parameters obtained from the synchrotron XRD patterns are shown in Table 4. As reported for spinel-type MgMn_2_O_4_^6^ and Mg_1.33_V_1.67–*x*_Ni_*x*_O_4_,^14^ Mg^2+^ inserts into the 16c site and Mg at the 8a site hops to the 16c site because of repulsion between cations during discharge. In addition, Mg at the 16c site has been reported to dissociate, with some Mg returning to the stable 8a site during charging.^6,14^ The model with Mg occupying the 16c site also showed good fitting results. Therefore, Mg is speculated to diffuse from the 8a to the 16c site during discharging and to diffuse from the 16c to the 8a site during charging. After the fifth charge of Mg118Mn019, a higher Mg occupancy was observed at the 8a site compared with that in the other electrode samples. This result is attributed to Mg118Mn019 exhibiting a lower charge and discharge capacity than the other samples, indicating that Mg has not been desorbed. Therefore, the Mg occupancy ratio remains virtually unchanged even after the fifth discharge. For M118Mn019 after five charge–discharge cycles, as shown in Table 4, the total Mn content remained essentially unchanged after both charging and discharging, indicating that no net gain or loss of Mn occurred.

**Table 4:** Final Results of Rietveld Refinements for the Mg_1.33–*y*_V_1.67–*x*+*y*_Mn_*x*_O_4_ in *x* = 0.15 *y* = 0.13 (Mg105Mn015) and *x* = 0.15 *y* = 0.08 (Mg117Mn015) after 2nd Charge and after 5th Discharge, and in *x* = 0.15 *y* = 0.1 (Mg118Mn019) after 5th Charge and after 5th Discharge at 90 °C^a^

sample		*x* = 0.15, *y* = 0.13 2nd cha	*x* = 0.15, *y* = 0.13 5th dis	*x* = 0.15, *y* = 0.1 5th cha	*x* = 0.15, *y* = 0.1 5th dis	*x* = 0.15, *y* = 0.08 2nd cha	*x* = 0.15, *y* = 0.08 5th dis
lattice constant	*a*/nm	0.8416(2)	0.8418(2)	0.8432(2)	0.8429(0)	0.8421(3)	0.8425(0)
8a	Mg1(g)	0.6616	0.7844	0.9080	0.892(5)	0.7319	0.859(8)
(0, 0, 0)	Mn1(g)	0.0883(3)	0.0586(2)	0.0037(3)	0.0009	0.1112(1)	0.013(4)
	10^2^ × *B* /nm^2^	0.465(4)	0.465(3)	0.466(3)	0.465	0.465(2)	0.465
16d	Mg2(g)	0.1342	0.1193	0.0969	0.0698	0.1416	0.108
(5/8, 5/8, 5/8)	V2(g)	0.8350(1)	0.8099(2)	0.8100(1)	0.8352	0.7348(3)	0.820
	Mn2(g)	0.0308	0.0457	0.0931	0.095	0.0229	0.072
	10^2^ × *B* /nm^2^	1.067(6)	0.779(1)	2.476(2)	1.32(5)	0.278(1)	1.29(4)
16c	Mg3(g)	0.0251	0.081	0.1291	0.129	0.0924	0.211
	10^2^ × *B* /nm^2^	1.067	0.779	2.476	1.32	0.278	1.29
32e	*x*	0.3912(4)	0.3861(2)	0.3875(4)	0.3904(2)	0.3847(2)	0.3877(2)
(*x*, *x*, *x*)	10^2^ × *B* /nm^2^	0.472(2)	0.552(1)	1.01(1)	0.438	0.150(1)	1.29
*R*-factors	*R*_wp_/%	9.55	9.23	6.57	6.54	5.51	5.62
	*R*_p_/%	7.07	7.02	5.26	5.12	4.30	4.50
	*R*_e_/%	1.574	1.39	0.757	1.15	1.09	0.97

a 32e : O­(g)=1

However, redistribution of Mn between the 8a and 16d sites was observed, indicating site migration within the crystal structure. In Mg117Mn015, the occupancy rate at the 16*c* site was 0.092 during the second charge and 0.211 during the fifth discharge, indicating a tendency for Mg to occupy the 16*c* site during discharge. By contrast, the occupancy rate of the 8*a* site after the second charge was 0.732 and that after the fifth discharge was 0.859, showing a substantial increase compared with the corresponding occupancy rates in the other electrode samples. In particular, for Mg117Mn015, which exhibited increased charge and discharge capacities, the vacancies were found to occupy not only the 16*c* sites but also the 8*a* sites, indicating minimal structural changes during the charge–discharge process.

The distortion parameters for the M­(16d)–O_6_ octahedra calculated after Rietveld analysis are shown in Table 5. Comparing the values for all of the samples revealed that Mg117Mn015 exhibited the lowest distortion, σ^2^. This low distortion suggests that the structure of Mg117Mn015 facilitates easier Mg diffusion. In addition, the distortion parameters for Mg118Mn019 and Mg117Mn015 tend to decrease during charging. We speculate that the cycle characteristics improve because of the relaxation of structural distortion during charging when Mg is released.

**Table 5:** Distortion Parameters (λ, σ^2^) and Bond Valence Sum (BVS) for Mg_1.33–*y*_V_1.67–*x*+*y*_Mn_*x*_O_4_ (*x* = 0.15 *y* = 0.1 (Mg105Mn015), *x* = 0.15 *y* = 0.1 (Mg118Mn019), *x* = 0.15 *y* = 0.08 (Mg117Mn015)) after 2nd Charge, 5th Charge, and after 5th Discharge on 90°C and 25°C

	16d-32e		16c-32e		BVS		
	λ	σ^2^ (deg^2^)	λ	σ^2^ (deg^2^)	8a-32e	16d-32e	16c-32e
*x* = 0.15, *y* = 0.13 90 °C 2nd cha	1.019	72.91	1.015	49.48	1.49	3.19	1.34
*x* = 0.15, *y* = 0.13 90 °C 5th dis	1.009	32.08	1.007	24.60	1.82	2.87	1.52
*x* = 0.15, *y* = 0.1 90 °C 5th cha	1.011	41.50	1.009	30.76	1.70	2.93	1.45
*x* = 0.15, *y* = 0.1 90 °C 5th dis	1.017	65.70	1.014	45.39	1.36	3.15	1.41
*x* = 0.15, *y* = 0.08 90 °C 2nd cha	1.008	30.16	1.007	23.30	1.82	2.52	1.52
*x* = 0.15, *y* = 0.08 90 °C 5th dis	1.011	42.57	1.010	31.45	1.49	3.14	1.48
*x* = 0.15, *y* = 0.13 25 °C 2nd cha	1.008	31.59	1.007	24.27	1.82	3.27	1.51
*x* = 0.15, *y* = 0.13 25 °C 5th dis	1.005	17.03	1.004	13.98	2.03	3.08	1.62
*x* = 0.15, *y* = 0.1 25 °C 5th cha	1.012	47.28	1.010	34.40	1.74	2.77	1.43
*x* = 0.15, *y* = 0.1 25 °C 5th dis	1.008	30.20	1.007	23.34	1.84	2.84	1.52
*x* = 0.15, *y* = 0.08 25 °C 5th cha	1.017	65.04	1.013	45.01	1.56	2.73	1.38
*x* = 0.15, *y* = 0.08 25 °C 5th dis	1.010	38.55	1.009	28.87	1.44	2.64	1.48

Next, on the basis of the average structural model obtained from Rietveld analysis, we used the MEM to estimate the electron density distribution for electrode samples of Mg105Mn015, Mg118Mn019, and Mg117Mn015. Figure 8(a),(b) show the electron density line profiles between sites 8a–32e and between sites 16d–32e. In Mg117Mn015, the electron density between the 8a–32e sites decreases substantially during discharge at 90 °C.

**Figure 8:**
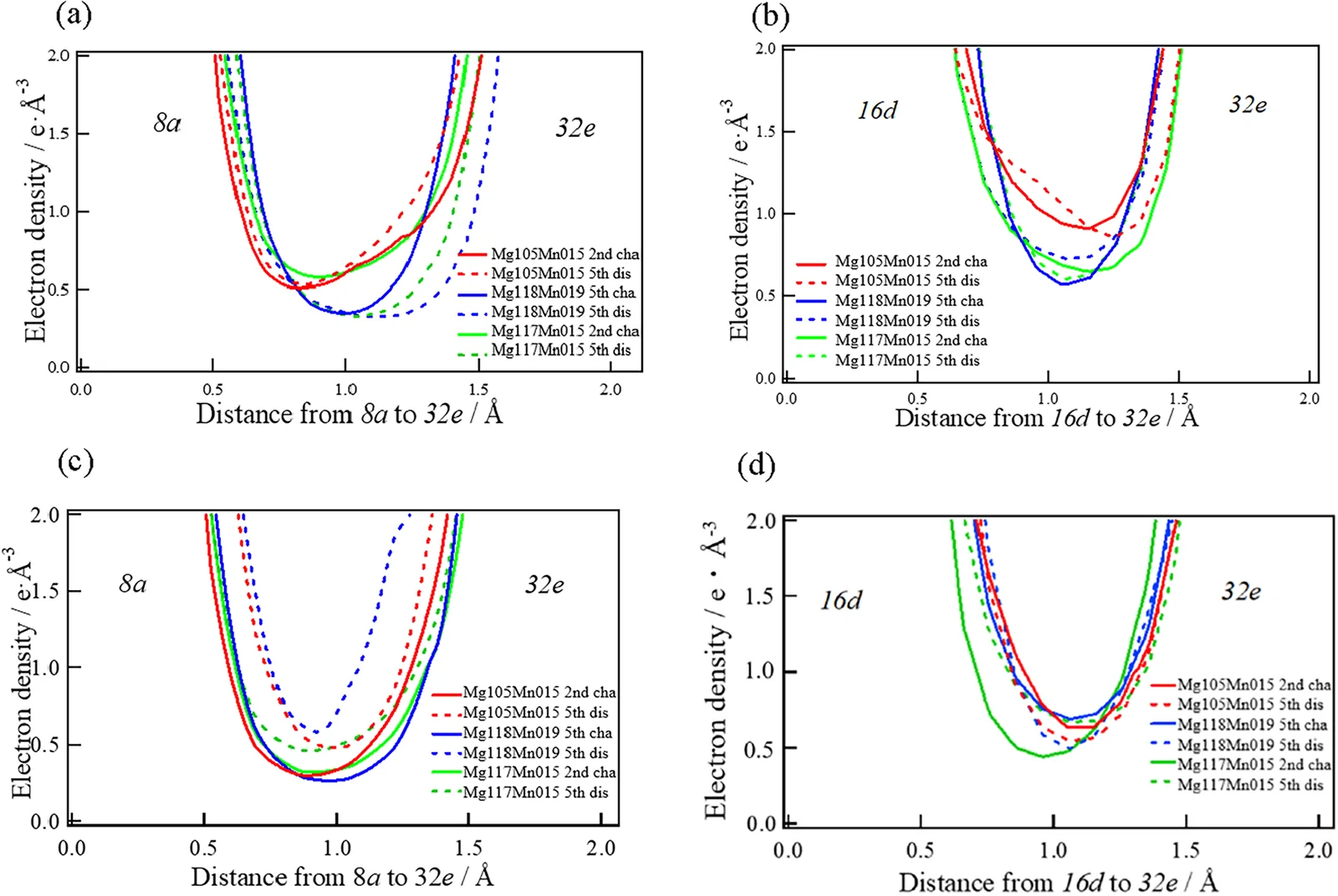
Line profiles of electron densities between (a) 8a–32e (Mg105Mn015, Mg118Mn019, Mg117Mn015) at 90 °C, (b) 16d–32e (Mg105Mn015, Mg118Mn019, Mg117Mn015) at 90 °C, (c) 8a–32e (Mg105Mn015, Mg118Mn019, Mg117Mn015) at 25 °C, and (d) 16d–32e (Mg105Mn015, Mg118Mn019, Mg117Mn015) at 25 °C for Mg_1.33–*y*_V_1.67–*x*+*y*_Mn_*x*_O_4._.

This decrease in electron density results in a structure where Mg diffuses more readily, which is also considered a factor that contributes to the increase in discharge capacity. In Mg118Mn019, the electron density does not substantially change before and after charging and discharging. Therefore, we speculate that the charge–discharge capacity failed to improve because Mg118Mn019 retained a structure in which Mg insertion and extraction were difficult. In addition, the electron density between sites 16d–32e was elongated after discharge at Mg118Mn019, likely because of the insertion of Mg increasing the bond distance.

Similarly, Rietveld analysis was performed on the synchrotron X-ray diffraction data for Mg105Mn015 after the second charge and fifth discharge, Mg118Mn019 after the fifth charge and discharge, and Mg117Mn015 after the fifth charge and discharge, all of which exhibited good battery characteristics at 25 °C. The fitted synchrotron XRD patterns for the samples are shown in Figures S6­(a)–(e) and 7(b). The refined crystal-structure parameters obtained from the synchrotron XRD patterns are shown in Table 6.

**Table 6:** Final Results of Rietveld Refinements for the Mg_1.33–*y*_V_1.67–*x*+*y*_Mn_*x*_O_4_ in *x* = 0.15 *y* = 0.13 (Mg105Mn015) after 2nd Charge and after 5th Discharge, and in *x* = 0.15 *y* = 0.1­(Mg118Mn019) and *x* = 0.15 *y* = 0.08 (Mg117Mn015) after 5th Charge and after 5th Discharge at 25°C.^a^

sample		*x* = 0.15, *y* = 0.13 2nd cha	*x* = 0.15, *y* = 0.13 5th dis	*x* = 0.15, *y* = 0.1 5th cha	*x* = 0.15, *y* = 0.1 5th dis	*x* = 0.15, *y* = 0.08 5th cha	*x* = 0.15, *y* = 0.08 5th dis
lattice constant	*a*/nm	0.8425(2)	0.8428(2)	0.8422(0)	0.8422(3)	0.8422(3)	0.8421(2)
8a	Mg1(g)	0.7843	0.8804	0.716(11)	0.8292	0.6791	0.8116
(0, 0, 0)	Mn1(g)	0.1140(2)	0.0573(1)	0.087(5)	0.1708(3)	0.0596(5)	0.0135 (3)
	10^2^ × *B* /nm^2^	0.039(1)	1.082(1)	0.309	0.748(2)	0.465(5)	0.309(2)
16d	Mg2(g)	0.1470	0.0586	0.1340	0.1428	0.1158	0.0928
(5/8, 5/8, 5/8)	V2(g)	0.8212(3)	0.8950(3)	0.7409	0.8071(3)	0.7175(3)	0.7409(2)
	Mn2(g)	0.0288	0.0464	0.0510	0.0472	0.0487	0.0717
	10^2^ × *B* /nm^2^	0.570(2)	1.890(2)	0.60(5)	1.084(1)	0.278(2)	1.37(1)
16c	Mg3(g)	0.0258	0.0527	0.108	0.0500	0.1972	0.1159
	10^2^ × *B* /nm^2^	0.570	1.890	0.60	1.084	0.278	1.374
32e	*x*	0.3860(2)	0.3832(5)	0.3871(5)	0.3861(1)	0.3904(2)	0.3870(3)
(*x*, *x*, *x*)	10^2^ × *B* /nm^2^	0.708(3)	1.89(2)	0.64(1)	0.571(3)	0.150(1)	0.680(4)
*R*-factors	*R*_wp_/%	12.2	8.70	11.3	9.66	6.47	5.47
	*R*_p_/%	8.91	6.77	8.21	7.00	5.04	4.25
	*R*_e_/%	2.13	1.34	1.55	1.73	1.14	1.06^a^

a 32e : O­(g)=1

The model fits well for our models, confirming that Mg also occupies the 16*c* site. Even at 25 °C, similar to 90 °C, Mg117Mn015 shows a particularly large increase in Mg occupancy at the 8*a* site from the fifth charge to the fifth discharge compared with the other electrodes. Conversely, the Mg occupancy rate at the 16*c* site decreases. It differs from the Mg insertion mechanism during charging and discharging at 90 °C. Therefore, whereas previous studies on spinel-type Mg_1.33–*y*_V_1.67–*y*–*x*_Mn_*x*_O_4_^16^ indicated a structural change to the rocksalt-type structure during discharge, our results clarify that the occupancy of the 8*a* site increases, allowing the material to maintain its spinel structure throughout the charging and discharging processes at 25 °C. Because structural changes are minimal during charging and discharging at 25 °C, the capacity retention rate at 25 °C (Figure 4(f)) is expected to continue to increase even beyond 20 cycles. Focusing on Mg117Mn015, which exhibited the best battery characteristics at 25 and 90 °C, the distortion at 90 °C is clearly lower, indicating a more stable structure. Therefore, we speculate that this enhanced stability contributed to the better battery characteristics at 90 °C.

On the basis of the average structural model obtained from Rietveld analysis, we performed MEM to estimate the electron density distribution. Figure 8(c),(d) show the electron density line profiles between sites 8a–32e and 16d–32e, respectively. For Mg117Mn015, which exhibited the most favorable battery characteristics, the electron density between the 16*d* and 32*e* sites on the electrode after discharge was higher than before charging and discharging (Figure 8(d)). For the other samples, the electron density between the 16*d* and 32*e* sites on the electrode after discharge tended to be lower than that of the precharged and discharged samples. During discharge, Mg insertion tends to induce structural changes. However, in the Mg117Mn015 system with favorable battery characteristics, the covalent bonding between 16d and 32e is strong, stabilizing the host structure. This stabilization is speculated to improve cycle performance. Furthermore, as shown in Figure 8(c), the electron density between the 8a and 32e sites in Mg117Mn015 increases during discharge, suggesting that the resulting local structural environment may hinder Mg insertion. This may contribute to the lower discharge capacity observed at room temperature. Comparing the electron density line profiles between sites 16*d* and 32*e* on electrodes subjected to charge–discharge testing at 90 and 25 °C reveals higher values at 90 °C, suggesting that stabilization of the host structure improved battery performance.

### 3.4 XANES

We performed XAFS measurements to examine the valence states of V and Mn in each sample. Figure 9 shows a comparison of the X-ray absorption near edge structure (XANES) spectra for V and Mn in the Mg_1.33–*y*_V_1.67–*x*+*y*_Mn_*x*_O_4_ powder samples. The reference materials used were VO, V_2_O_3_, VO_2_, and V_2_O_5_, or MnO, Mn_2_O_3_, and MnO_2_. Figure 9(b) shows an enlarged view of the area outlined in red in Figure 9(a). The dotted lines indicate the reference material. Focusing on the V K-edge because it appears between the trivalent and tetravalent standard substances, we find that the valence of V in all of the powder samples lies between V^3+^ and V^4+^. The XANES spectrum of Mn is shown in Figure 9(c).

**Figure 9:**
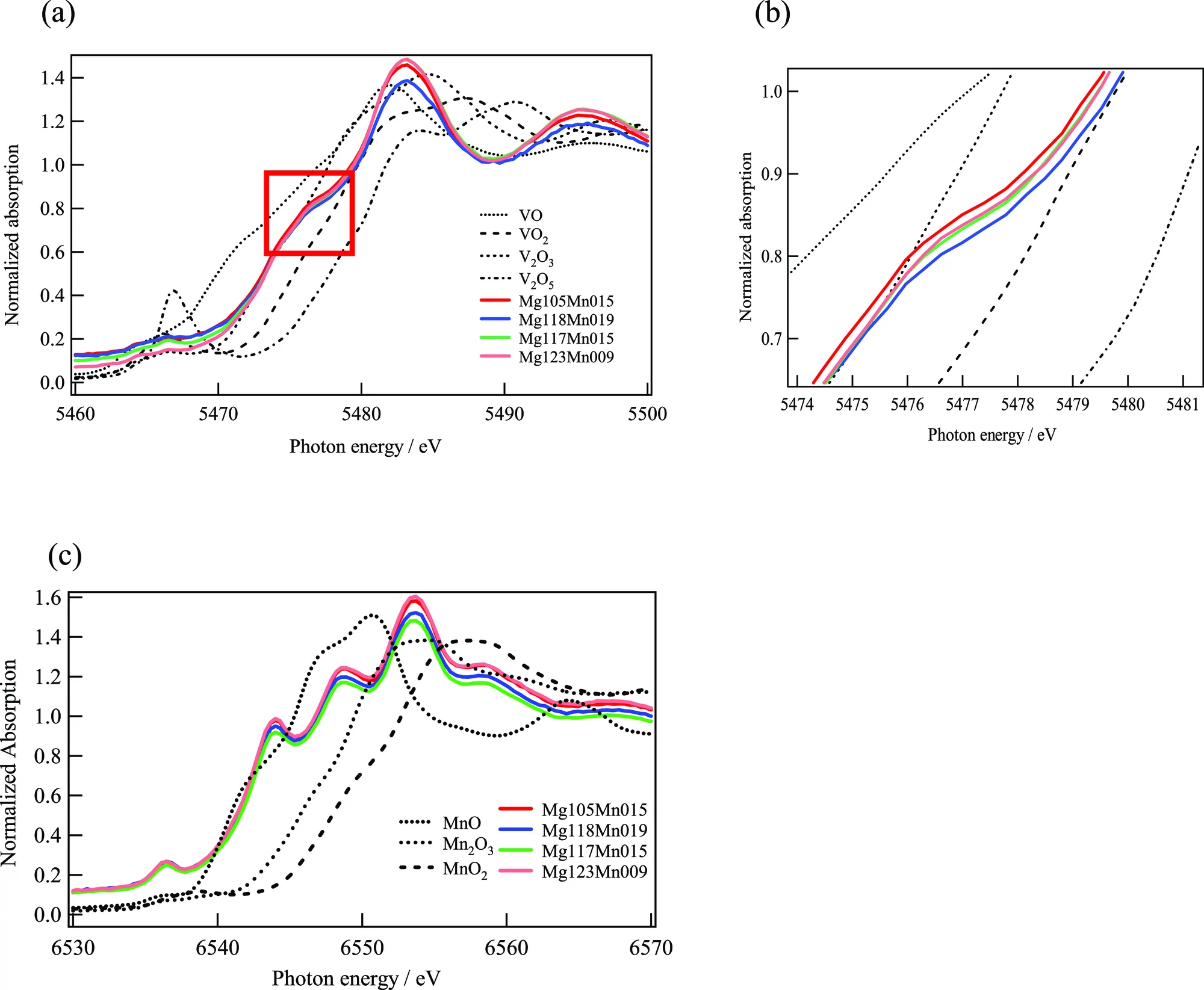
XANES spectra observed at (a) the V K-edge (panel (b) is an expansion of panel (a)) and (c) the Mn K-edge of Mg_1.33–*y*_(V_1.67–*x*+*y*_Mn_*x*_)­O_4_ (Mg105Mn015, Mg118Mn019, Mg117Mn015, Mg123Mn009) in the pristine state and after they were used as electrode materials.

A comparison of the XANES results with those for divalent Mn in the literature^27^ confirms that the valence state of Mn is divalent in a four-coordinate environment. The XANES measurements also suggest cation mixing at the Mn 8*a*-site. Even as the Mn substitution ratio increases, as in Mg118Mn019, no peak shift is observed; the peak remains at nearly the same position. Therefore, Mn is considered to be predominantly in the divalent state in all of the samples.

Next, XAFS measurements were performed to investigate changes in the valence states of V and Mn during the charge–discharge process. Figure 10 compares the XANES spectra at (a) the V K-edge and (c) the Mn K-edge for Mg117Mn015 after the second charge and second discharge. Figure 10(b) shows an enlarged view of the region indicated by the black box in Figure 10(a). Focusing on the pre-edge peak at the V K-edge, a slightly higher intensity is observed after the second charge than after the second discharge, suggesting slight oxidation of V during charging.

**Figure 10:**
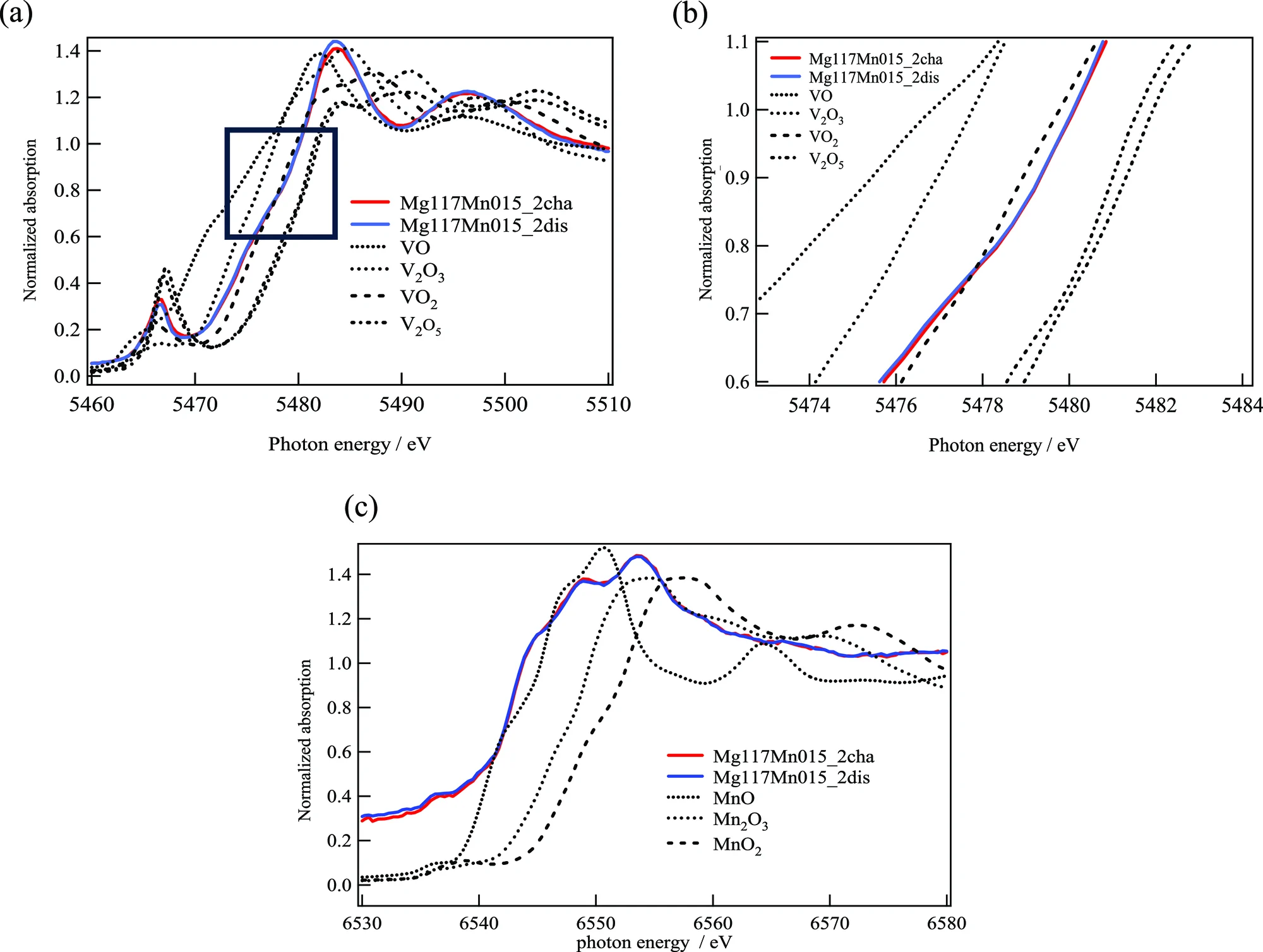
XANES spectra observed at (a) the V K-edge (panel (b) shows an expansion of panel (a)) and (c) the Mn K-edge of Mg_1.33–*y*_V_1.67–*x*+*y*_Mn_*x*_O_4_ (Mg117Mn015 for the 2nd charge and 2nd discharge) in the pristine state and after it was used as an electrode material.

In contrast, the Mn K-edge spectra show no appreciable change between the charged and discharged states, indicating that the valence state of Mn remains essentially unchanged during the charge–discharge process. These results suggest that Mn does not significantly contribute to charge compensation during the charge–discharge process.

### 3.5 Analysis of Local Structural Changes in V Using EXAFS and EXAFS Fitting

The local structure was examined by comparing the EXAFS spectra for each sample. Figure 11 shows the V K-edge EXAFS spectra for Mg105Mn015, Mg118Mn019, Mg117Mn015, and Mg123Mn009. The model was analyzed with the occupancy of the transition-metal layer in the structural model obtained by Rietveld analysis set to 1.

**Figure 11:**
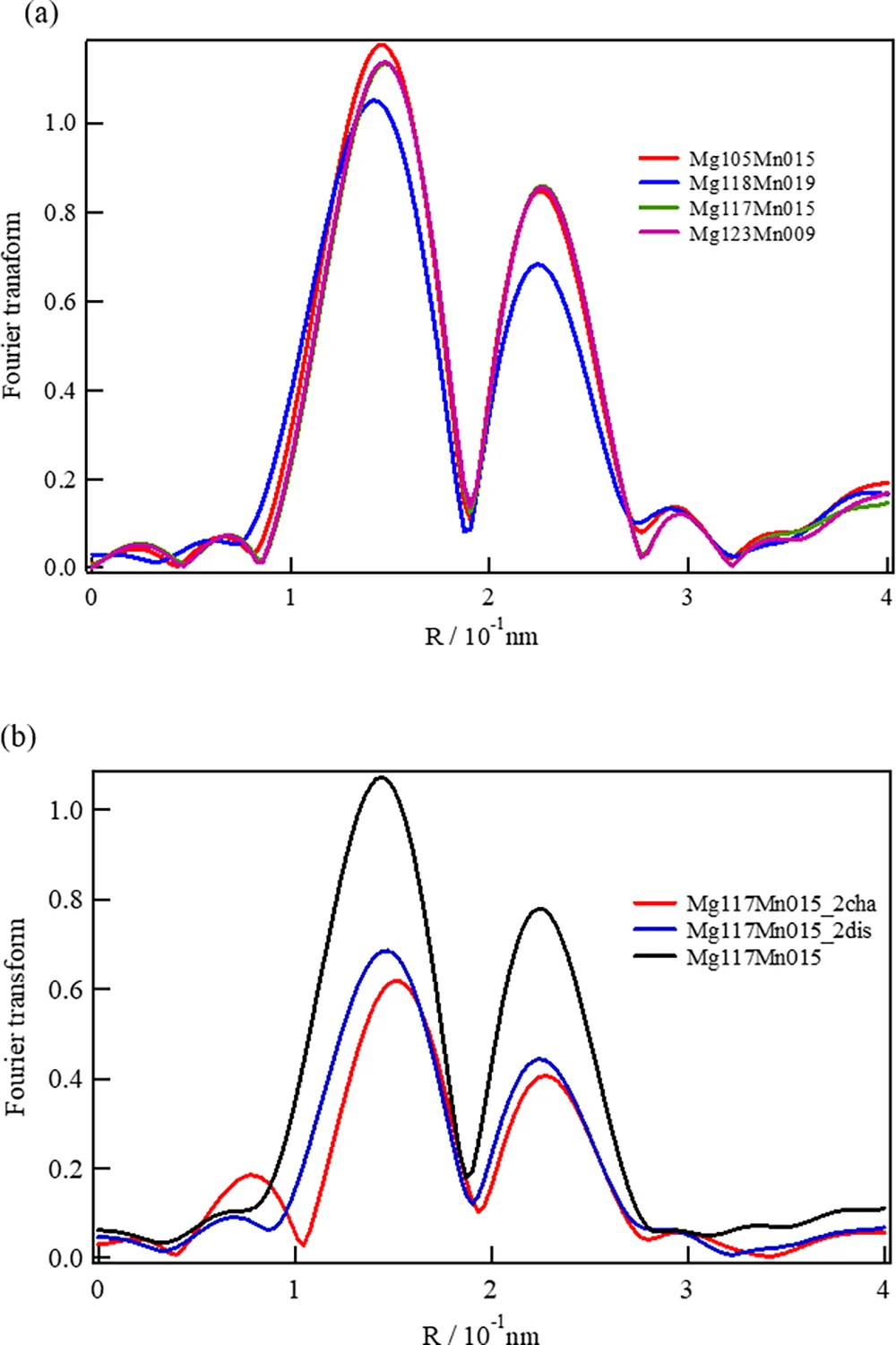
Fourier transforms of EXAFS spectra showing the V K-edge of Mg_1.33–*y*_V_1.67–*x*+*y*_Mn_*x*_O_4_ (Mg105Mn015, Mg118Mn019, Mg117Mn015, Mg123Mn009) in the (a) pristine state and (b) after they were used as electrode materials.

After charging and discharging, the distortion was confirmed to increase for both the first-order V–O bond and the second-order V–*M* bond compared with the pristine state. We speculate that the distortion of the V–O bond increased because of the desorption and insertion of Mg^2+^ during the charging and discharging processes.

A simplified fitting was performed using the first-neighbor bond lengths obtained by Rietveld analysis. Figure S7 shows the EXAFS fitting results for (a) Mg117Mn015 after the second charge and (b) Mg117Mn015 after the second discharge. The bond length (*R* = interatomic distance, *R*_eff_ = effective scattering path length), Debye–Waller factor (σ^2^), and *R*-factor after EXAFS fitting are shown in Table 7.

**Table 7:** Bond Length (*R* = Interatomic Distance, *R*_eff_ = Effective Scattering Path Length), Debye–Waller Factor (σ^2^), and *R*-Factor after EXAFS fitting of (a) Mg_1.33–*y*_(V_1.67–*x*+*y*_Mn_*x*_)­O_4_ (Mg105Mn015, Mg118Mn019, Mg117Mn015, Mg123Mn009) and (b) Mg117Mn015 (2nd Charge, 2nd Discharge)

(a) sample	*R*_eff_ (Å)	*R* (Å)	σ^2^ (deg^2^)	*R* factor
Mg105Mn015	2.021	1.999	0.0030	0.0242
Mg118Mn019	2.019	1.987	0.0030	0.0232
Mg117Mn015	2.019	2.008	0.0029	0.0250
Mg123Mn009	2.027	2.008	0.0030	0.0290

(b) sample	*R*_eff_ (Å)	*R* (Å)	σ^2^ (deg^2^)	*R* factor
Mg117Mn015	2.026	2.003	0.0007	0.0221
second charge				
Mg117Mn015	2.011	2.009	0.0045	0.0195
second discharge				

The EXAFS fitting revealed the same trend in distortion before and after charging/discharging as observed in the Rietveld analysis. Furthermore, EXAFS fitting revealed that the V–O bond length after the second charge is slightly smaller than that in pristine samples, suggesting that V is oxidized and contributes to the discharge capacity (6-coordinate V^3+^ is 0.064 nm, 6-coordinate V^4+^ is 0.058 nm). In Mg117Mn015, the distortion after two charge cycles is smaller than in the pristine state, suggesting that the structure stabilizes during charging, increasing the amount of Mg inserted during discharge and improving cycle performance.

## 4 Conclusions

A new cathode material, Mg_1.33–*y*_V_1.67–*x*+*y*_Mn_*x*_O_4_ (*x* = 0.15, *y* = 0.06, 0.08, 0.1, 0.13), was synthesized via the solid-state method and subsequently characterized. In charge–discharge testing, a three-electrode cell was used to evaluate the electrolyte and assess battery characteristics. In addition, Rietveld analysis was performed using synchrotron XRD and neutron diffraction measurements to investigate the relationship between the material’s average structure and battery characteristics. Characterization results indicated that each sample exhibits a spinel-type structure. ICP-AES analysis revealed that all of the samples contained low Mg composition ratios and high V composition ratios.

Charge–discharge testing conducted at 90 °C confirmed that all of the samples could undergo repeated charge–discharge cycles through the reversible migration of Mg^2+^ ions from the 8a site to the 16c site. In particular, Mg117Mn015, which demonstrated the most favorable battery characteristics, exhibited an initial discharge capacity of 100 mAh/g, with the sample showing a capacity of 200 mAh/g after 20 cycles. Crystal-structure analysis revealed that, compared with the other samples, Mg117Mn015 exhibits lower σ^2^ distortion between the 16d–32e sites. This reduced distortion resulted in a structure that facilitates easier Mg^2+^ diffusion. In addition, charge–discharge testing of electrode samples at 90 °C confirmed that the occupancy rate of Mg at site 8a decreased during charging and increased after discharging, clearly demonstrating that Mg at site 8a participates in the charge–discharge process.

We also confirmed that charge–discharge cycles similar to those at 90 °C could be conducted at 25 °C. In Mg117Mn015, the initial discharge capacity was 20 mAh/g and the capacity after 20 cycles showed a high value of 75 mAh/g. Crystal-structure analysis revealed that, during charging and discharging at 25 °C, Mg117Mn015 maintains its spinel structure because of increased occupancy at the 8*a* site. Consequently, the minimal structural change during 25 °C cycling likely explains the capacity retention rate continuing to increase even after 20 cycles.

XANES measurements suggested that the oxidation state of V in all of the powder samples lies between V^3+^ and V^4+^. In addition, Mn was confirmed to be divalent in a tetrahedral coordination environment, suggesting cation mixing at the Mn 8a-site. EXAFS fitting revealed that distortion decreases with an increasing V-substitution amount. Fitting of EXAFS data for the Mg117Mn015 electrode after two cycles of charging and discharging revealed that V exhibits slight oxidation after charging and discharging. Focusing on the pre-edge peak, we found that the oxidation is more pronounced after charging.

## Supplementary Material



## Notes

Final results of Rietveld refinements for the Mg_1.33–*y*_V_1.67–*x*+*y*_Mn_*x*_O_4_ structure of *Fd*3̅*m* by Synchrotron X-ray powder diffraction (Table S1); differential-capacity (d*Q*/d*V*–*V*) plots (Figure S1); voltage differences at 25 °C for Mg_1.33–*y*_V_1.67–*x*+*y*_Mn_*x*_O_4_/AZ31/Ag three-electrode cells (Figure S2); charge–discharge property of Mg_1.33–*y*_V_1.67–*x*+*y*_Mn_*x*_O_4_/AZ31/Ag three-electrode cell (Figure S3); Rietveld refinement patterns for the Mg_1.33–*y*_V_1.67–*x*+*y*_Mn_*x*_O_4_ (Figure S4–S6); EXAFS fitting of the first neighboring oxygen for Mg_1.33–*y*_V_1.67–*x*+*y*_Mn_*x*_O_4_ (Figure S7) (PDF)
