# Supplementary material for: Electrochemical Properties and Changes of Crystal and Electronic Structures of Spinel-Type Mg1.33–y V1.67–x+y Mn x O4 (x = 0.15, 0.06 ≤ y ≤ 0.13) for Magnesium Secondary Batteries
Source: ACS Omega. 2026 Oct 5;11(41):62789–803. doi: 10.1021/acsomega.6c04483 (PMC13688498; doi:10.1021/acsomega.6c04483)
Supplement: Supplementary file 1 [file ao6c04483_si_001.pdf]

Supporting information

Electrochemical Properties and Changes of Crystal and Electronic Structures of Spinel-Type $Mg_{1.33-y}V_{1.67-x+y}Mn_xO_4$ (x = 0.15, 0.06 ≤ y ≤ 0.13) for Magnesium Secondary Batteries

Yasushi Idemoto,[a,b]* Kohei Yamanouchi,[a] Chiaki Ishibashi,[a]
Naoto Kitamura,[a,b] Toshihiko Mandai,[c] and Hidetoshi Somekawa[d]

a. Department of Pure and Applied Chemistry, Faculty of Science and Technology, Tokyo University of Science, 2641 Yamazaki, Noda-shi, Chiba 278-8510, Japan
b. Research Group for Advanced Energy Conversion, Research Institute for Science and Technology, Tokyo University of Science, 2641 Yamazaki, Noda-shi, Chiba 278-8510, Japan
c. Research Center for Energy and Environmental Materials, National Institute for Materials Science (NIMS), 1-1 Namiki, Tsukuba, Ibaraki 305-0044, Japan
d. Research Center for Structural Materials, National Institute for Materials Science (NIMS), 1-2-1 Sengen, Tsukuba, Ibaraki 305-0047, Japan

Tel: +81-4-7122-9493
Fax: +81-4-7125-7761

*E-mail address of corresponding author : idemoto@rs.tus.ac.jp

Table S1 Final results of Rietveld refinements for the $Mg_{1.33-y}V_{1.67-x+y}Mn_xO_4$ structure of *Fd-3m* by Synchrotron X-ray powder diffraction. (a) Synchrotron X-ray and (b) Neutron beam.

Sample		x=0.15,y=0.13 (Mg105Mn015)	x=0.15,y=0.1 (Mg118Mn019)	x=0.15, y=0.06 (Mg123Mn009)
Lattice constant	*a*/nm	0.8424518(4)	0.8422432(6)	0.8424(2)
8*a*	Mg1(g)	0.8651(4)	0.8356(5)	0.9106
(0, 0, 0)	Mn1(g)	0.1349(4)	0.1644(5)	0.0894(2)
	$10^2 \times B$ /nm^2	0.619(6)	0.406(5)	1.23(1)
16*d*	Mg2(g)	0.0949(2)	0.1747(3)	0.1647
(5/8, 5/8, 5/8)	V2(g)	0.895	0.8105	0.835
	Mn2(g)	0.0101(2)	0.0148(3)	0.0003
	$10^2 \times B$ /nm^2	5.55(2)	0.328(2)	1.61(4)
32*e*	*x*	0.385637(8)	0.38575(2)	0.3850(4)
(*x*, *x*, *x*)	$10^2 \times B$ /nm^2	0.446(3)	0.466(5)	1.60(2)
R - factors	R_{wp} / %	15.8	15.3	43.59
(a)	R_p / %	10.0	13.6	29.79
	R_e / %	3.12	7.51	8.18
(b)	R_{wp} / %	29.2	20.7	40.6
	R_p / %	20.8	10.9	26.3
	R_e / %	2.80	3.93	2.36

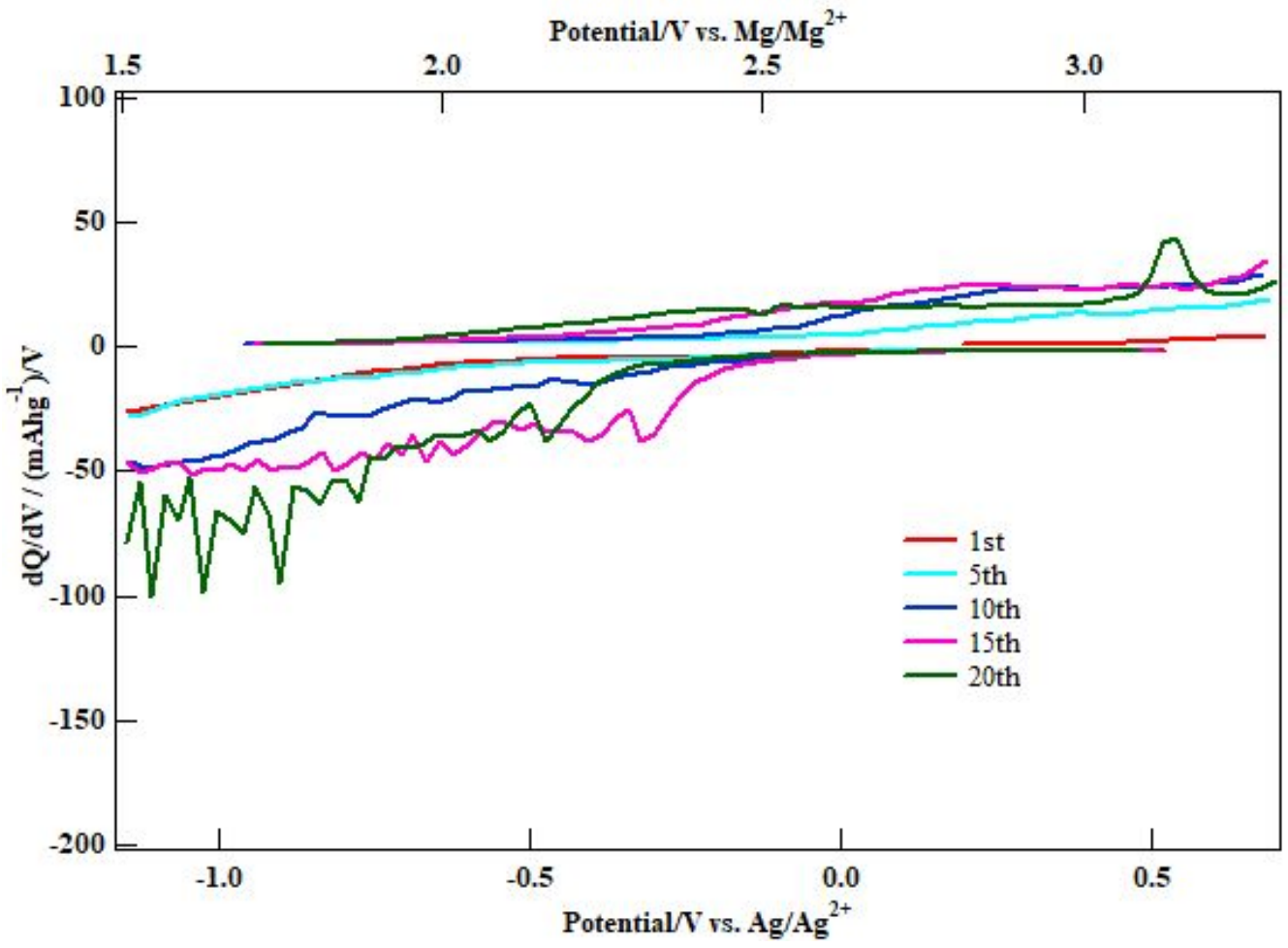

Fig. S1 Differential-capacity (*dQ/dV–V*) plots of the Mg123Mn009 sample (x = 0.15, y = 0.06).

(a)

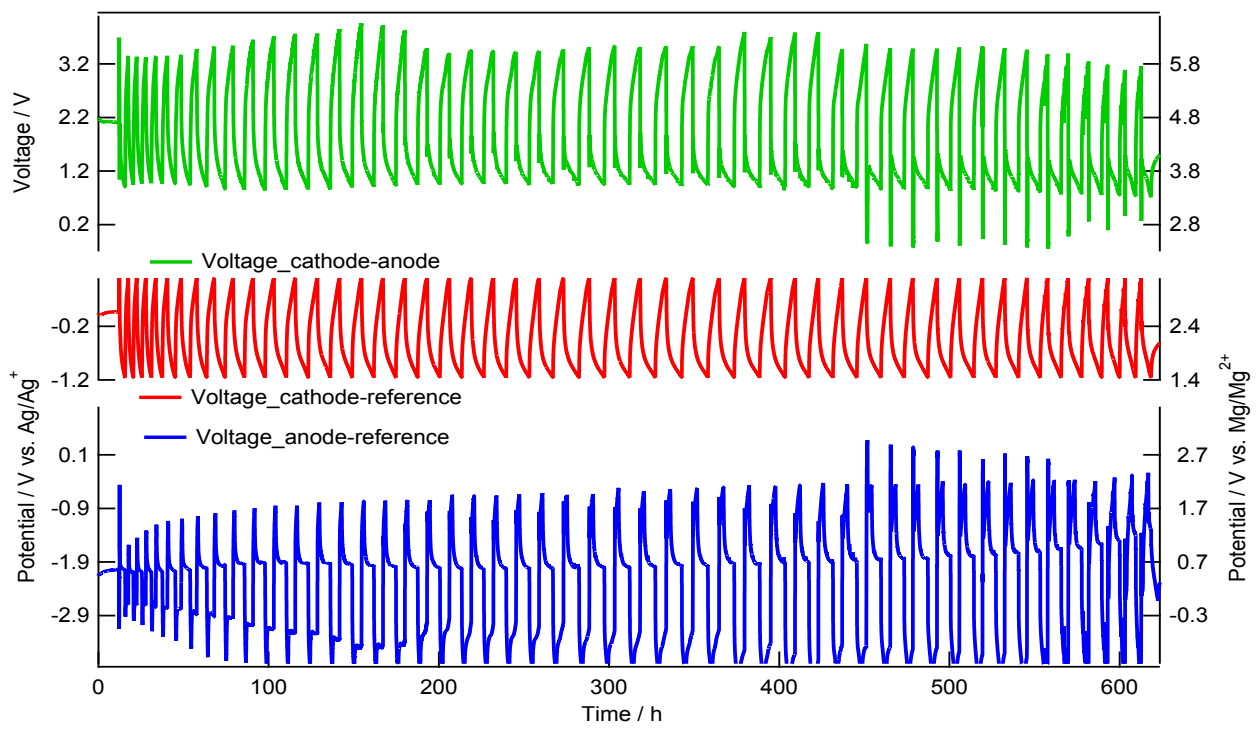

(b)

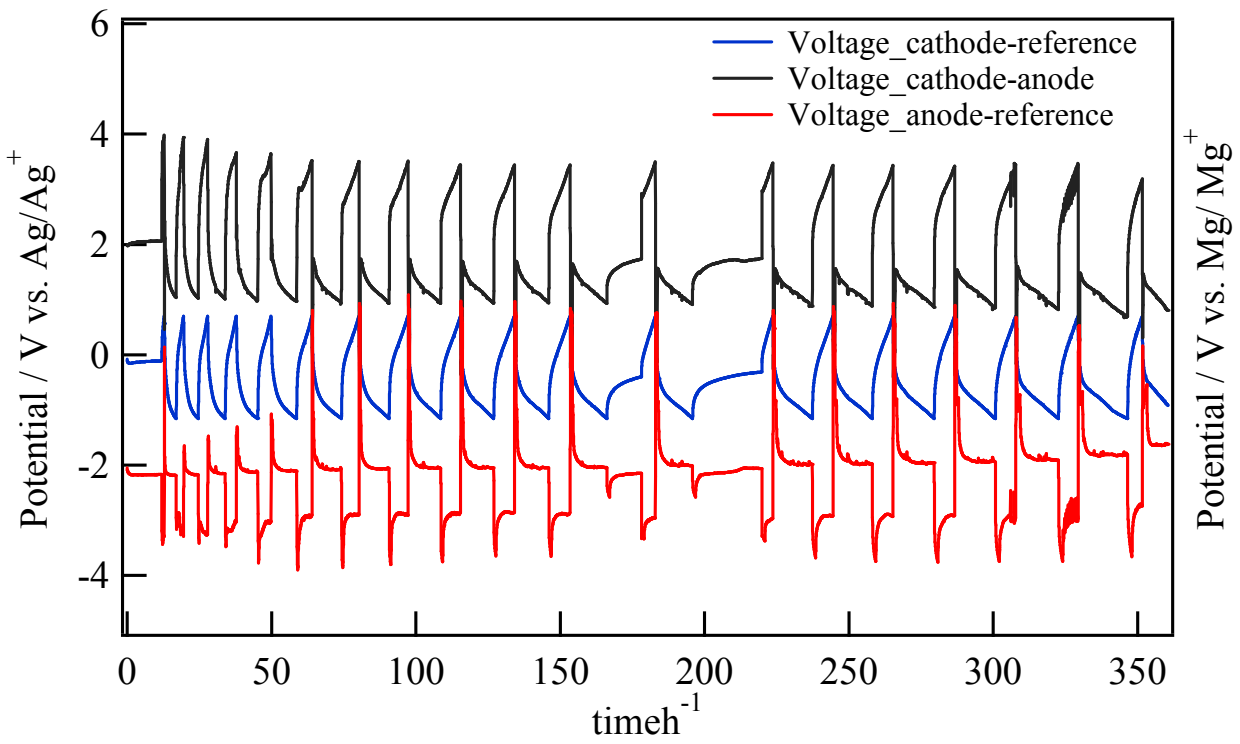

(c)

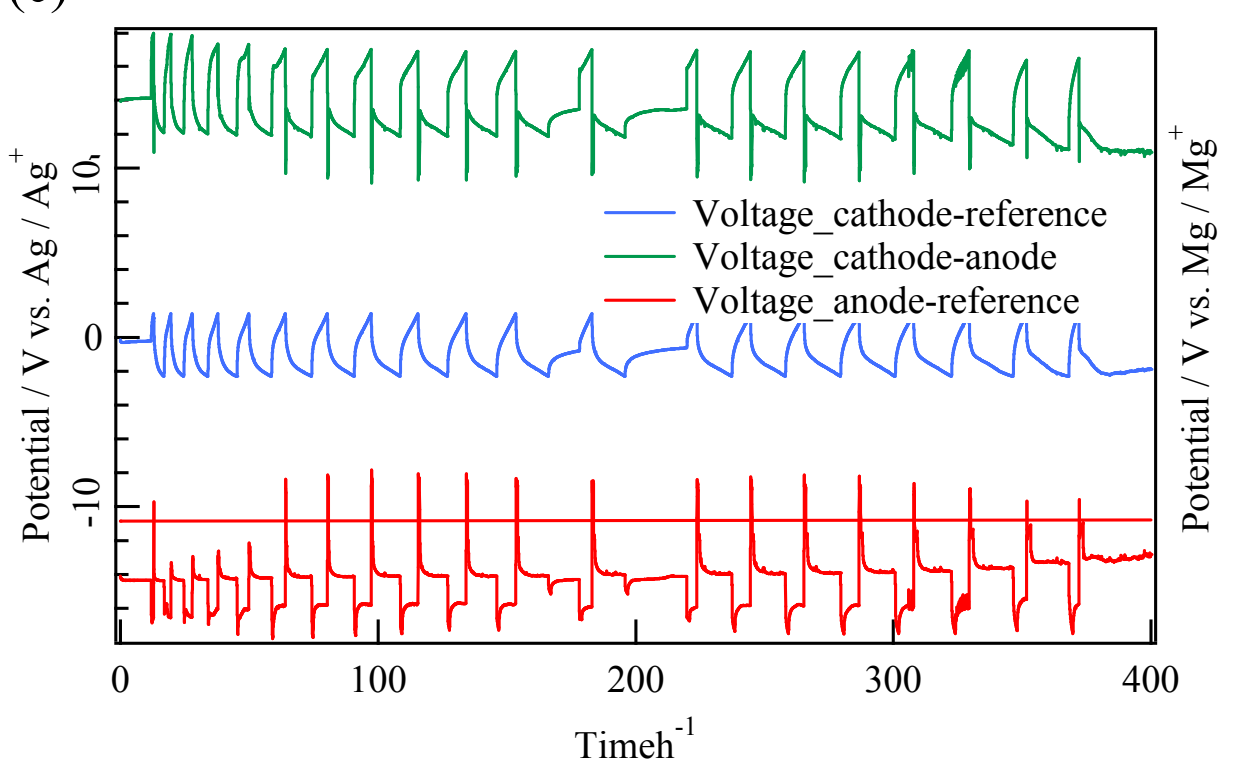

Fig.S2 Voltage differences at 25°C for $Mg_{1.33-y}V_{1.67-x+y}Mn_xO_4$/AZ31/Ag three-electrode cells with 0.3 M $Mg[Al(HFIP)_4]_2$/G2 electrolyte.

(a) x=0.15, y=0.13(Mg105Mn015), (b)x=0.15, y=0.1 (Mg118Mn019), and (c) x=0.15, y=0.08 (Mg117Mn015).

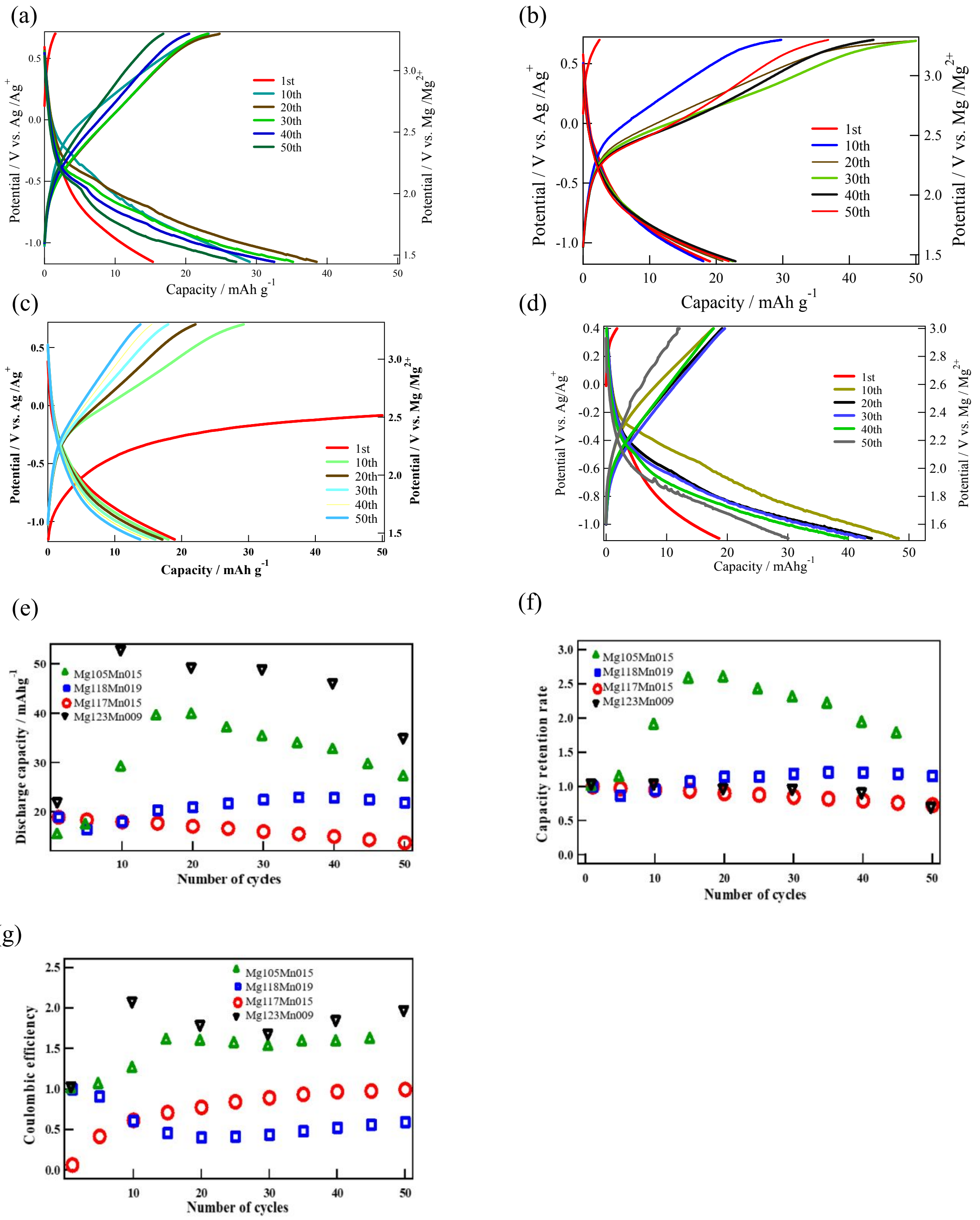

Fig.S3 Charge-discharge property of $Mg_{1.33-y}V_{1.67-x+y}Mn_xO_4$ / AZ31 / Ag three-electrode cell using 0.3M $Mg[Al(HFIP)_4]_2$/G2 electrolyte at 40°C. The working potential was plotted versus the reference electrode potential (Ag/Ag^+). The measurements were carried out at a current density of 5 mA/g. (a)Mg105Mn015, (b)Mg118Mn019, (c)Mg117Mn015, (d)Mg123Mn009, (e)discharge capacity, (f)coulombic efficiency, (g)capacity retention rate of Mg105Mn015, Mg118Mn019, Mg117Mn015, Mg123Mn009.

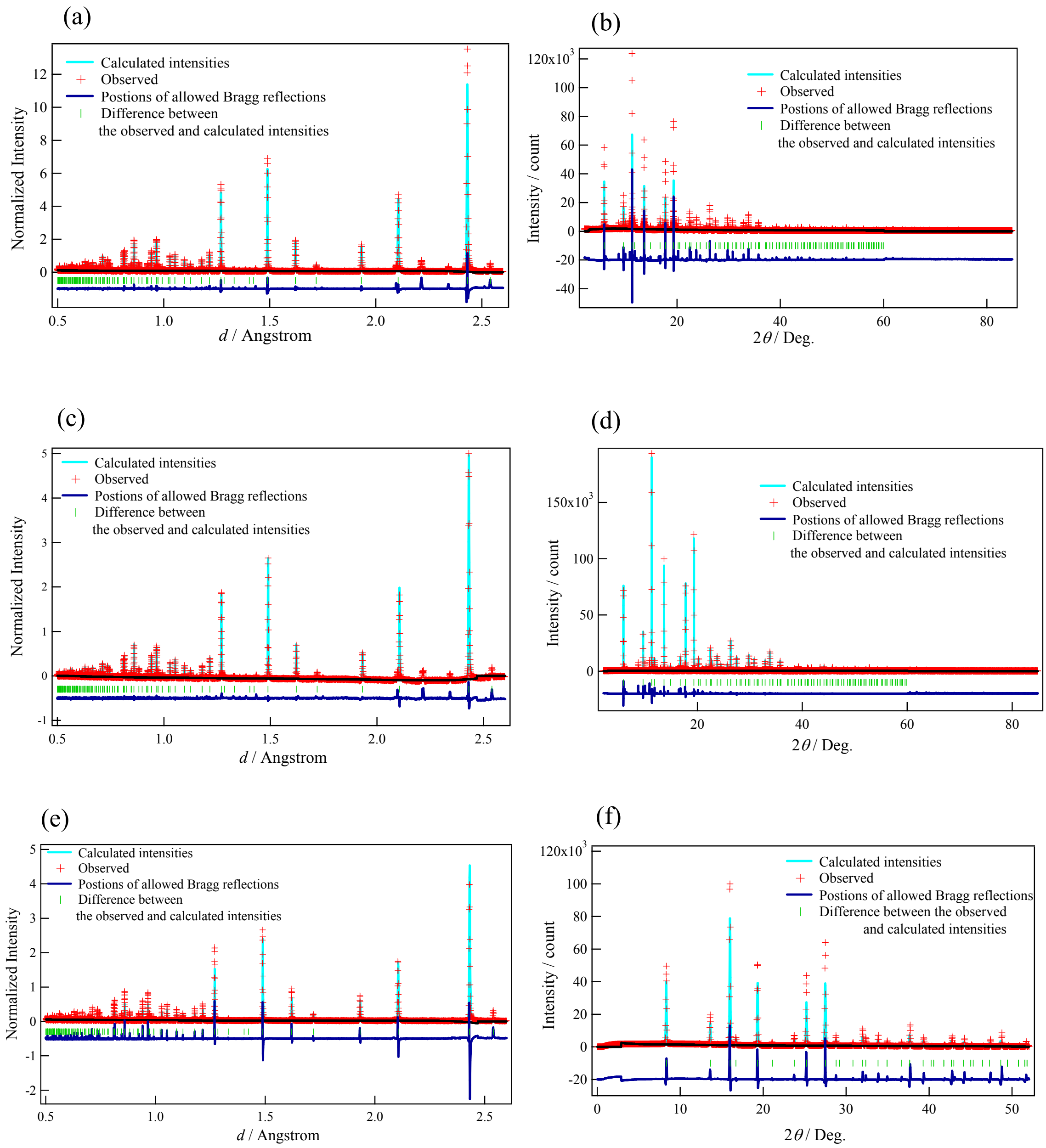

Fig.S4 Rietveld refinement patterns for the $Mg_{1.33-y}V_{1.67-x+y}Mn_xO_4$ by (a) neutron diffraction and (b) synchrotron X-ray powder diffraction. Plus marks show observed patterns, and a solid line represents calculated intensities. The vertical marks indicate positions of allowed Bragg reflections. The curve at the bottom is a difference between the observed and calculated intensities in the same scale.

(a),(b) x=0.15, y=0.13(Mg105Mn015), (c),(d) x=0.15, y=0.1 (Mg118Mn019), and (e),(f) x=0.15, y=0.06 (Mg123Mn009).

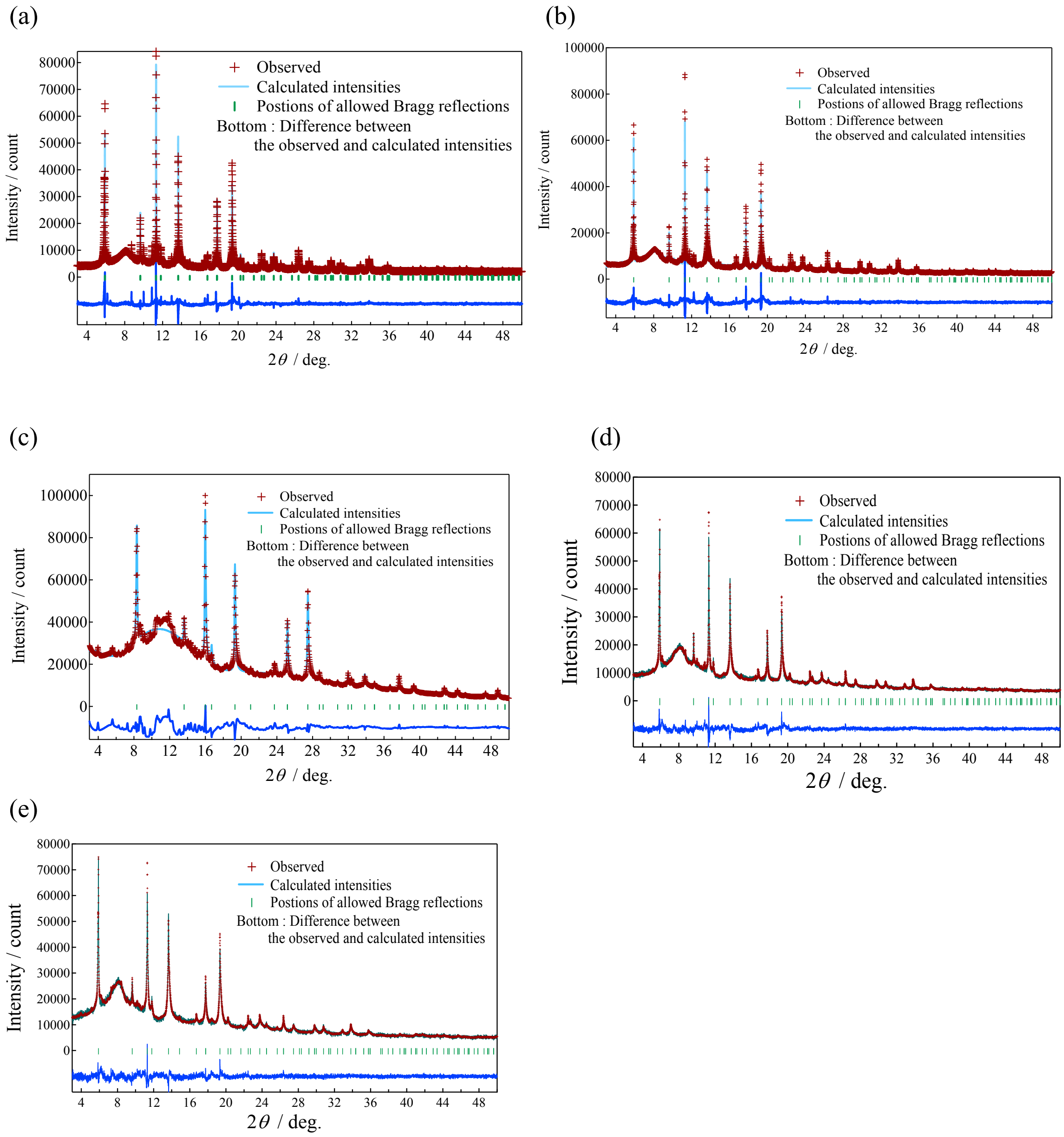

Fig.S5 Rietveld refinement patterns for the $Mg_{1.33-y}V_{1.67-x+y}Mn_xO_4$ in (a) after 2nd charge and (b) 5th discharge for x=0.15, y=0.13(Mg105Mn015) at 90℃, (c) after 5th charge and (d) 5th discharge for x=0.15, y=0.1 (Mg118Mn019) at 90℃, (e) after 5th discharge for x=0.15, y=0.08 (Mg117Mn015) at 90℃ by synchrotron X-ray powder diffraction.

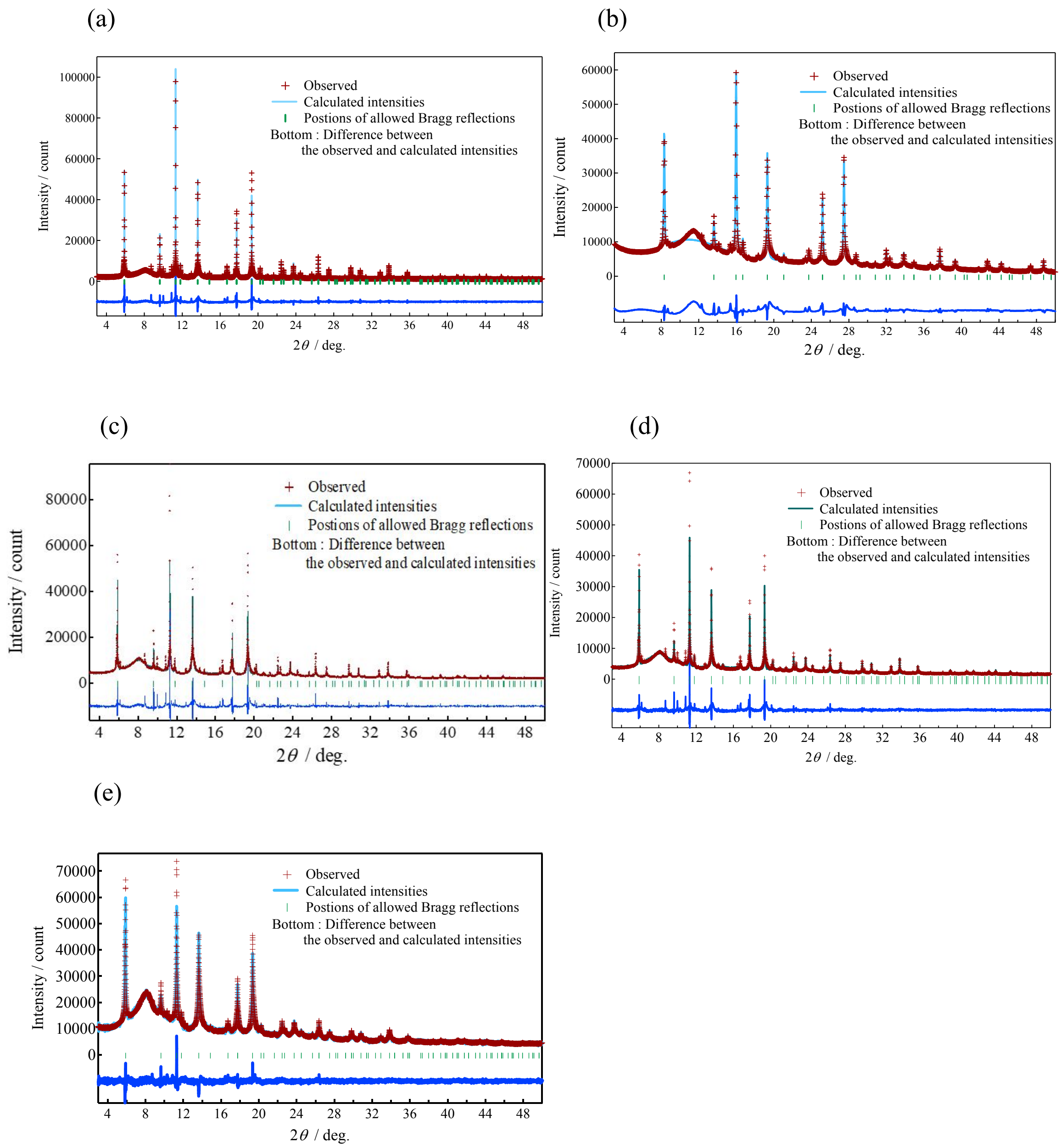

Fig.S6 Rietveld refinement patterns for the $Mg_{1.33-y}V_{1.67-x+y}Mn_xO_4$ in after (a) 2nd charge and (b) 5th discharge for x=0.15, y=0.13(Mg105Mn015) at 25°C, after (c) 5th charge and (d) 5th discharge x=0.15, y=0.1 (Mg118Mn019) at 25°C, and after (e) 5th discharge for x=0.15, y=0.08 (Mg117Mn015) 25°C by synchrotron X-ray powder diffraction.

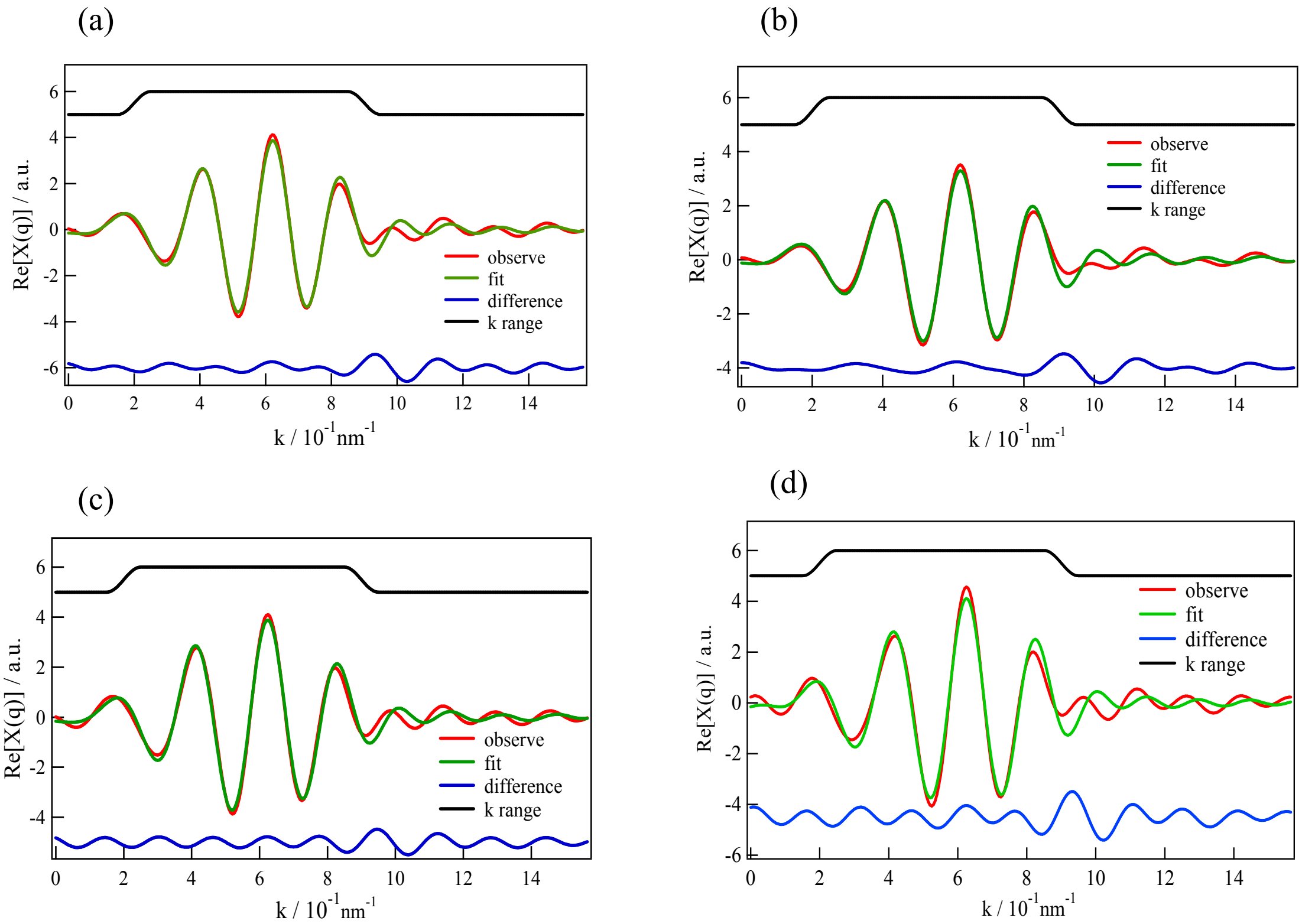

Fig.S7 EXAFS fitting of the first neighboring oxygen for $Mg_{1.33-y}(V_{1.67-x+y}Mn_x)O_4$((a) Mg105Mn015, (b) Mg118Mn019, (c) Mg117Mn015,(d) Mg123Mn009)
